# An integrated approach to uncertainty and global sensitivity analysis in penstock structural modeling

**DOI:** 10.1016/j.heliyon.2024.e41049

**Published:** 2024-12-12

**Authors:** Manal Haddouch, Imane Hajjout, El Mostapha Boudi

**Affiliations:** a Department of Mechanical Engineering, Mohammadia School of Engineering, Avenue Ibn Sina B.P 765, Agdal, Rabat, 10090, Morocco; b Renewable Energies Laboratory, Energy and Farm Machinery Department, Hassan-II Agronomic and Veterinary Institute, Madinat Al Irfane, Rabat, 6202, Morocco

**Keywords:** Penstock modeling, Modal analysis, Uncertainty analysis, Regional sensitivity analysis, Latin hypercube sampling, PAWN method

## Abstract

Enhanced penstock structural models significantly advance hydropower engineering, yet their increasing complexity introduces challenges. As model interactions intensify, predictability and comprehensibility decrease, complicating the evaluation of model accuracy and alignment with operational performance metrics and safety standards. This issue is particularly pronounced in dynamic modeling, where knowledge gaps hinder straightforward validation via observational data. Traditional techniques for model calibration and validation are becoming impractical, necessitating a strategic approach to prioritize sources of uncertainty related to critical response variability. This study aims to advance our understanding and management of structural variabilities in penstock models by developing a comprehensive, step-by-step Global Sensitivity Analysis (GSA), designed to meet the specific needs of penstock modeling. Illustrated through a free vibration analysis model of a penstock span, this structured methodology begins with Uncertainty Analysis (UA) to identify variabilities, followed by a screening phase using the Morris method to enhance computational efficiency. Subsequent application of multi-method GSA ranks parameter sensitivities, assesses robustness, and provides a comparative evaluation, providing insights into the effective tools for conducting GSA on penstock models. The process culminates with Regional Sensitivity Analysis (RSA), targeting local sensitivities and enhancing understanding of local parameter influences, thereby supporting model adjustments and design optimization. Results from this application characterize model sensitivities for the most prominent mode shapes to specific structural parameters and indicate that these sensitivity outcomes are influenced by variability in parameter spaces and output sub-range definitions. This study provides a practical framework for addressing uncertainties in penstock design, enhancing model accuracy, prioritizing parameters, managing risks, and improving the reliability and efficiency of hydropower infrastructure.

**Table:** Nomenclature

UQ	Uncertainty Quantification	EEs	Elementary Effects
UA	Uncertainty Analysis	OAT	One-At-Time
GSA	Global Sensitivity Analysis	FAST	Fourier Amplitude Sensitivity Test
SA	Sensitivity Analysis	EFAST	Extended FAST
LSA	Local Sensitivity Analysis	RBD	Random Balance Design
FF	Factor fixing	PDF	Probability Density Function
FP	Factor prioritization	ECDFs	Empirical Cumulative Density functions
FM	Factor mapping	DMIM	Delta Moment Independent Measure
RSA	Regional sensitivity analysis	MPF	Mode Participation Factor
LHS	Latin Hypercube Sampling	KDE	Kernel Density Estimation
MCF	Monte Carlo Filtering	FEM	Finite Element Modeling

## 1 Introduction

In addressing the multifaceted challenges facing the global energy sector, including volatile energy prices, fluctuating construction costs, and increasing global energy demand, engineers are tasked with designing infrastructures that are economically efficient, adaptable, and safe. Central to hydroelectric power generation, penstocks are crucial components in hydropower systems, serving as conduits that channel high-pressure water to turbines, thus playing a pivotal role in energy conversion efficiency. Ensuring the structural integrity of penstocks is vital for the stability and safety of power systems, as any compromise can lead to significant financial, safety, and environmental risks.

Current penstock design methodologies primarily rely on deterministic models, which inadequately address the uncertainties inherent in real-world hydropower systems. These uncertainties, stemming from factors such as variability in material properties, fluctuating hydraulic loads, and changing operational conditions, introduce significant and often underestimated risks, especially as the use of lighter, cost-effective materials in penstocks becomes more prevalent in penstocks.

Moens et al. [1] emphasize the importance of accounting for uncertainties in structural dynamics, advocating for their integration into reliability and durability assessments to mitigate potential safety and performance concerns. Similarly, Xu et al. [2] underscore the necessity of understanding these uncertainties for accurate modeling and stability evaluation in hydraulic generating systems.

Uncertainties also arise from modeling assumptions and modelers perspectives [3,4], that can distort predictive accuracy, as highlighted by Hornberger and Spear [5] and Pfleiderer [6]. The lack of observational data presents a supplementary source of uncertainty, which poses significant risks of structural failure [7]. Accurate evaluation of penstock models requires analyzing their stability under diverse conditions such as varying hydraulic loads and dynamic responses. Designers must have access to relevant data to adeptly manage interdisciplinary and conflicting objectives.

A transition to a probabilistic design approach, specifically Uncertainty Quantification (UQ), is crucial for addressing these uncertainties more effectively and ensuring the long-term reliability and robustness of penstock structures [6,8]. UQ broadly represents the science of characterizing uncertainty in system outcomes and involves the quantitative assessment of uncertainties in system behavior. A key technique within UQ is Sensitivity Analysis (SA) [9], which helps identify the factors contributing most to uncertainty, aiding decision-makers in managing risk and improving design choices [10].

Global Sensitivity Analysis (GSA) extends this by evaluating the influence of input uncertainties across the entire parameter space, and capturing interaction effects between variables. GSA is particularly suited for complex systems, as it provides a comprehensive view of how variations in multiple factors collectively impact the system's response, helping prioritize the most influential parameters for robust decision-making [[11], [12], [13], [14]]. Seminal works [[15], [16], [17]] offer deeper insights into the integration of UQ and GSA, highlighting their value for improving the reliability of complex systems under uncertainty.

Despite the growing recognition of its benefits in modeling processes [18,19], the application of SA across various technical fields, including hydropower systems and infrastructures, remains underutilized. Saltelli et al. [20] attribute this to several factors, such as the neglect of model uncertainties, an emphasis on methodological approaches over clearly defined objectives [21,22], and the lack of interdisciplinary collaboration.

Historically, most use of sensitivity analysis for structural models have predominantly relied on Local sensitivity analysis (LSA), particularly derivative-based methods that are suitable for well-defined functional forms with limited input variability. These methods, commonly integrated with shape optimization in linear models, use local slope estimations to assess sensitivity around optimal parameter sets [23]. Techniques like the boundary element method and isogeometric analysis have been utilized within this framework [24,25].

Specifically for penstock modeling, Zhou et al. [26] explored sensitivity analysis to optimize an internal stiffener plate in a penstock bifurcation, examining how the moving boundary affects stress constraints and identifying key design parameters impacting structural integrity. Similarly, Haddouch et al. [27] used the One-At-Time (OAT) method, also a form of LSA, to analyze the impact of individual structural parameters to stresses and mass in exposed penstock models, highlighting critical parameters for design optimization.

Probabilistic methods have also been explored, as demonstrated by Bryla et al. [28], who assessed penstock integrity by quantifying the impact of manufacturing variations and corrosion. They employed methods such as the First-Order Reliability Method (FORM) to evaluate failure probabilities based on key parameters like residual wall thickness and material properties. Ajenjo et al. [29] further extended this work by combining info-gap decision theory with line sampling to evaluate penstock reliability under epistemic uncertainty, providing insights into optimization efforts. On the maintenance side, Çelikdemir [30] used parametric sensitivity analysis on a proposed equation model to assess how changes in various parameters influence maintenance costs in small hydroelectric power plants.

These techniques are classified as LSA methods, which are widely recognized as valuable tools, particularly for linear models with limited input variability. However, LSA has been criticized for failing to fully capture parameter importance in non-linear models with complex interactions [31]. Additionally, LSA's limited exploration of parametric space can introduce biases in non-linear models [32,33].

The advent of Global Sensitivity Analysis (GSA) marks a methodological shift toward a more comprehensive assessment of parameter influences on complex model behavior.

In the context of penstock modeling, the aim of GSA is to bridge the knowledge gap regarding the impact of uncertainties on penstock systems. By addressing these uncertainties and the complexities of their structural integrity, GSA enables a more accurate evaluation of design risks, leading to better-informed decisions during both the design and maintenance phases of penstock systems.

However, the application of GSA in this field is hindered by the high computational cost and complexity associated with large-scale, non-linear models [18].

In response to these gaps, the contribution of this paper is threefold: i. Advanced modeling of structural uncertainties by incorporating UA techniques, with the goal of addressing the uncertainties and complexities inherent in their structural integrity of the penstocks. ii. Structured and practical framework for conducting global sensitivity analysis (GSA) on penstock models, aimed at addressing the challenges associated with GSA application in penstock. The proposed framework integrates UA and provides structured guidance for selecting and applying the most appropriate GSA methodologies. iii. The utility of the proposed GSA framework is demonstrated through a Practical Implementation involving the modal analysis of a pre-stressed penstock span, showcasing how this approach can be used to optimize penstock designs.

The following sections will outline the step-by-step methodology, demonstrate its utility through a pre-stressed modal analysis of a penstock span model, and discuss how these findings can be applied to enhance the design and operational reliability of hydropower infrastructure.

## 2 Research methodology

Beyond the basic classification into LSA and GSA, Saltelli et al. [15] categorize SA into three distinct settings based on the purposes of the analysis: (i) Factor prioritization (FP); (ii) Factor fixing (FF); and (iii) Factor mapping (FM). The review by Wei et al. [34] comprehensively compares techniques of sensitivity analysis, referred to as variable importance analysis, and provides framework for selecting optimal techniques and best practices for SA in computational models, enhancing the accuracy and reliability of such analyses.

This section outlines the proposed systematic workflow for applying SA on penstock systems, designed to tackle the discussed challenges of high dimensionality, complexity, and non-linearity. In addressing these challenges, the selection of sensitivity analysis (SA) methods is driven by several key factors: The specific objectives of the analysis, the computational resources available, and the characteristics of the model itself. As penstock models often involve numerous input factors, it is crucial to choose methods that can balance the trade-off between computational cost and the accuracy of the results. Given the high dimensionality, the number of model evaluations (N) tends to increase with the number of input factors (k). However, this relationship is method-dependent, and different SA methods offer varying degrees of efficiency in handling large k/N ratios. The Morris method is computationally efficient for screening a large number of inputs, making them suitable as an initial step in a sequential approach. By reducing the number of influential factors, more computationally intensive methods, particularly the variance-based approaches, known for their accuracy (such Extended Fourier Amplitude Sensitivity Test (EFAST) method) can then be applied to a smaller subset of inputs, optimizing both accuracy and computational effort (Campolongo et al., 2011). In addition, the non-linearity in input-output relationships and the distributional properties of the model's outputs (e.g., skewness) must also be taken into account in selection of SA methods. Certain methods are more adept at capturing non-linear interactions or skewed output distributions. In this case, applying multiple GSA methods may increase the robustness of the results, as different methods can provide complementary insights (e.g., statistical methods for linear dependencies, variance-based methods for capturing parameter interactions, and moment independent methods for non-linearities). Lastly, scalability and computational efficiency were key considerations in our methodology. The methods selected are designed to handle the increasing complexity and size of penstock systems while ensuring that the analysis remains computationally feasible. By strategically selecting and combining methods, this phased methodology aimed to provide a comprehensive yet efficient sensitivity analysis framework tailored to the specific challenges of penstock modeling.

**Fig. 1:**
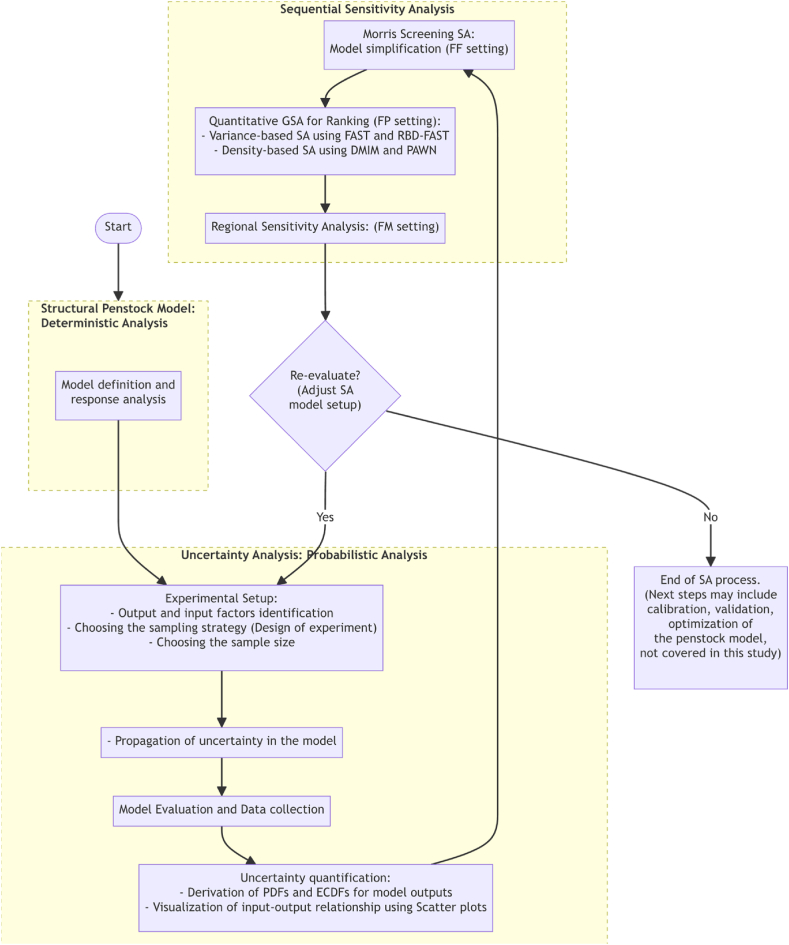
Step-by-step workflow for conducting Uncertainty analysis and GSA on penstock models.

As depicted in Fig. 1, this workflow initiates with UA (Sect. *2.1*) which propagates and estimates output variability, setting the stage for a goal-oriented SA framework. This includes screening analysis for FF purpose (Sect. *2.2*), to streamline the model for the computational tractability of subsequent quantitative methods, followed by GSA for FP purpose (Sect. *2.3*) to identify the main drivers of output variability. The process culminates with RSA for FM purpose (Sect. *2.4*), pinpointing the ranges of uncertain factors affecting specific model behaviors, critical for effective optimization and decision-making.

The workflow is inherently iterative, allowing adjustments based on insights gained in the final phase. By identifying and excluding parameter sub-ranges that lead to undesirable responses, the process refines the experimental setup and may revise the input variability space. This iterative nature ensures that the SA remains dynamically responsive to new findings, enabling more precise and impactful analyses.

### 2.1 Uncertainty analysis

Addressing the uncertainties and complexities of penstock design necessitates incorporating uncertainty modeling for informed decision-making. In this process, modeling response uncertainty, which involves a comprehensive exploration of input uncertainties, precede the sensitivity analysis. This practice, known as forward UQ [21], involves propagating input uncertainties through the model to anticipate uncertainties in the design process. It specifies the probabilities and shapes of distributions to characterize the probabilistic response of all factors of interest [35,36].

The objective is to guide the framing of the SA problem, select appropriate SA techniques, and refine response probability estimates. Recognizing these probabilities is the first step toward creating more robust designs, reducing risks, and making better-informed decisions.

This probabilistic modeling approach addresses the inherent unpredictability arising from the interplay of variables, maintaining its relevance despite the deterministic nature of the primary physical processes dictating the response. Attempting to accurately represent every deterministic scenario is often unfeasible due to prohibitive costs.

### 2.2 Factor screening

The second step of our workflow addresses the computational challenges inherent in analyzing penstock systems, which typically involve extensive models with numerous inputs. This phase capitalizes on the potential of SA for model simplification through the FF setting, which streamlines the model by omitting less relevant components, without notably affecting the output's variance. Such simplification does create a more manageable model for subsequent step by narrowing down the analysis to the most influential factors, thereby enhancing overall model efficiency and setting the stage for a more detailed GSA.

For this purpose, the screening design, particularly relevant for FF purposes. Several screening designs have been proposed in the literature as reviewed in Ref. [37]. The method of Morris and its extended version [38,39] offer several advantages over other screening methods. These include computational efficiency, broad applicability, flexibility, independence from model specifics, and freedom from restrictive assumptions [15].

Following the notation by Ref. [19], this method calculates global sensitivity using the mean of the EEs (finite differences) of each parameter at different locations, as shown in Eq. (1):

(1) $$S_{i}=\mu_{i}^{*}=\frac{1}{r}\sum_{j=1}^{r}EE_{i}^{j}=\frac{1}{r}\sum_{j=1}^{r}\frac{g\left(x_{1},\ldots ,{\bar{x}}_{i}+\Delta_{i},\ldots ,{\bar{x}}_{N}\right)-g\left({\bar{x}}_{1},\ldots ,{\bar{x}}_{i},\ldots ,{\bar{x}}_{N}\right)}{\Delta_{i}}c_{i}$$

with r is the number of trajectories (sample repetitions) in the input space, typically ranging from 4 to 10 [40], each $x_{j}$ denotes the points along each trajectory, where j=1,…,r, selected using the OAT sampling strategy. This method also quantifies parametric interactions by calculating the standard deviation of the EEs, as shown in Eq. (2):

(2) $$\sigma_{i}=\sqrt{\frac{1}{r}\sum_{j=1}^{r}{\left(EE_{i}^{j}-\frac{1}{r}\sum_{j=1}^{r}EE_{i}^{j}\right)}^{2}}$$

Higher values of $\sigma_{i}$ indicate that responses at different levels of factor $x_{i}$ vary significantly, suggesting substantial interactions between this factor and other uncertain factors.

### 2.3 Factor Prioritization

Following the initial screening to reduce the number of parameters, our methodology proceeds to the Factor Prioritization stage. This phase employs various GSA methods that considers multiple measures to capture the nuanced influences of different structural parameters. GSA aims to examine the full uncertainty ranges of the parameters within the model, offering a comprehensive view of their influence on the model's output by varying them concurrently [37].

The family of GSA methods includes non-parametric techniques, variance-based techniques, and moment-independent techniques. Reviews of these techniques are found in Ref. [41]. Indicators developed for GSA purposes are termed importance measures [34,42] or uncertainty importance measures [9,12,42,43] to distinguish them from local importance indicators [[44], [45], [46]] and screening methods.

GSA techniques are based on different assumptions and have been shown to produce varied sensitivity estimates [47]. Therefore, to ensure robust conclusions from the sensitivity analysis, it is advisable to employ multiple GSA methods whenever feasible. This approach not only corroborates the findings through diverse analytical perspectives but also enhances the reliability of the prioritization process. The following sections detail the appropriate techniques suitable for our types of penstock models.

This approach corroborates findings through diverse analytical perspectives and enhances the reliability of the prioritization process. In this context, we have selected two categories of techniques deemed appropriate for structural behavior analysis of penstocks.

#### 2.3.1 Variance decomposition methods

Variance-based SA methods assume that different model factors contribute differently to the variation of model outputs, thus decomposing and analyzing the output variance can determine a model's sensitivity to input parameters [40,48]. The design to compute these indices is based on Monte Carlo or some form of stratified sampling, such as for instance the Latin Hypercube Sampling (LHS) [11,49], with Sobol’ sequences [50,51] showing superior performance [52].

The first-order sensitivity index indicates the percent of model output variance contributed by a factor individually and is obtained using the following [48,53]:

(3) $$S_{i}=\frac{V_{X_{i}}\left(E_{X_{∼i}}\left(Y|X_{i}\right)\right)}{V\left(Y\right)}$$

where $X_{i}$ is the i-th factor and $X_{∼i}$ denotes the matrix of all factors but $X_{i}$. E and V denoting the expected value and the variance, respectively. The total SA index represents the entire influence of an input factor on model outputs including all of its interactions with other factors [52,54]. In other words, total-order indices include first-order and all higher-order interactions associated with each factor and can be estimated calculated using the following:

(4) $$S_{T_{i}}=\frac{E_{X_{∼i}}\left(V_{X_{i}}\left(Y|X_{∼i}\right)\right)}{V\left(Y\right)}=1-\frac{V_{X_{∼i}}\left(E_{X_{i}}\left(Y|X_{∼i}\right)\right)}{V\left(Y\right)}$$

Both $S_{i}$, $S_{T_{i}}$ have an intuitive interpretation. Referring to the numerators in Eqs. (3), (4) above, $V_{X_{i}}\left(E_{X_{∼i}}\left(Y|X_{i}\right)\right)$ is the expected reduction in variance that would be obtained if $X_{i}$ could be fixed, and $E_{X_{∼i}}\left(V_{X_{i}}\left(Y|X_{∼i}\right)\right)$ is the expected variance that would be left if all factors but $X_{i}$ could be fixed.

Variance is highly recommended as a summary measure of uncertainty whenever the application allows it [15]. Variance-based methods, such as Sobol’ indices [31,48,53] and the FAST [55], are particularly resource-intensive and entail extensive computational costs, especially when simulating large models like those involving penstock structures. The FAST method relies on the Fourier transform to decompose the variance of a model output y, providing an efficient way to measure main effects only [56]. For efficient management of computational resources, we recommend the hybrid version; Random Balance Designs-FAST (RBD-FAST), when only the first-order indices are needed [57,58]. RBD-FAST extends the FAST method by utilizing a random permutation procedure for sampling, improving robustness and accuracy. It can leverage any pre-existing sampling such LHS utilized during the UA or Morris sampling, thus avoiding redundant computational efforts and information loss. When capturing total effects, the EFAST [59] is more suitable and remains computationally more efficient than Sobol [37,59]. However, it is still necessary to perform factor screening analysis beforehand for model reduction when computing variance-based measures. The choice between the FAST variants hinges on the trade-off between cost and the required precision of the analysis.

#### 2.3.2 Moment-independent methods

Transitioning to density-based sensitivity methods, which arose from the works of Liu et al. [60] and Borgonovo et al. [61], provides a valuable alternative when variance is not an adequate proxy for uncertainty due to skewed or multi-modal distributions of the outputs. This approach evaluates the impacts across the entire output distribution rather than focusing only on variance, which is particularly relevant for non-linear models. Sensitivity in this context is related to the variations in the output Probability Density Function (PDF) that occur when the uncertainty about one input is removed. Unlike variance-based methods that emphasize output variance, density-based methods are moment-independent, determining sensitivity without relying on specific distribution assumptions. This characteristic is especially advantageous in systems where extreme events are critical. Additionally, these methods can utilize existing data samples without demanding unique sampling requirements.

Within this category, the Delta Moment-Independent Measure (DMIM) [62,63] and the PAWN [64,65] method stand out as particularly effective for penstock models. The DMIM considers different conditioning values of inputs $x_{i}$, and measures δ-sensitivity using the average area enclosed between the conditional probability density function (PDF) $f_{y|x_{i}}\left(y\right)$ and the unconditional PDF $f_{y}\left(y\right)$, as shown in Eq. (5):

(5) $$\delta_{i}=\frac{1}{2}E_{x_{i}}\left[\int_{-\infty }^{+\infty }\left|f_{y}\left(y\right)-f_{y|x_{i}}\left(y\right)\right|\;dy\right]$$

Conversely, the PAWN method differs from DMIM and other density-based methods by using the empirical cumulative distribution functions (ECDFs), instead of the PDFs, simplifying numerical implementation. Additional advantages of PAWN are that it can be easily tailored to focus on output sub-ranges such as extreme values, and that intermediate results generated in the application of PAWN can be visualized to gather insights about the model behavior, which can be used later for factor mapping. The logic behind this technique consists of assessing the distance between the unconditional ECDFs and the conditional ECDFs of the output when $x_{i}$ has been fixed; this distance accounts for the variability of the output that has been reduced due to fixing variable $x_{i}$, providing an importance measure of $x_{i}$ on the output. The PAWN sensitivity index for the i-th input factor is defined as shown in Eq. (6):

(6) $$S_{i}={\text{stat}}_{x_{i}}\;\text{KS}\left(x_{i}\right)\;\text{where}\;\text{KS}\left(x_{i}\right)=\max_{y}|F_{y}\left(y\right)-F_{y|x_{i}}\left(y|x_{i}\right)|$$

Here, $F_{y}\left(y\right)$ and $F_{y|x_{i}}\left(y|x_{i}\right)$ are the unconditional and conditional ECDFs of the output y, and stat is a user-defined statistic (e.g., maximum, median, or mean). The Kolmogorov-Smirnov (KS) statistic measures the distance between ECDFs. Other statistics, like the Anderson-Darling statistic, can also be used.

Both DELTA and PAWN benefit from utilizing pre-existing samples, as they do not require specifically tailored experimental designs. This enhances cost efficiency compared to variance-based methods.

### 2.4 Factor mapping

In the final phase of our SA workflow, we tackle the Factor Mapping through RSA [66] to achieve a refined understanding of model behavior, crucial for making precise optimization decisions. This advanced stage builds on the foundational work completed during the UA, factor screening, and GSA phases, setting the stage for a more targeted exploration.

RSA, also known as Regional Response Probabilistic SA [60], focuses on assessing model sensitivity within specific regions of the parameter space, particularly near critical design criteria, rather than across the entire model domain. By identifying zones where parameters have a heightened impact, RSA facilitates pinpointing necessary adjustments to optimize model responses, a key advantage for design engineers who face complex, localized decision-making challenges.

At the core of RSA, the process of ‘Factor Mapping’ addresses essential questions such as ‘Which regions in the parameter space significantly affect the model response?’ and ‘Which parameters induce specific responses within certain ranges?’ This phase is dedicated to examining local sensitivities and mapping the parameter space, thereby providing targeted guidance for parameter selection and system configuration. Additionally, RSA plays a key role in identifying non-identifiable parameters by analyzing local sensitivities across different output zones. Therefore, RSA can reveal the influence of factors previously deemed insignificant, either confirming their lack of impact or establishing their subtle yet critical roles.

By positioning RSA as the concluding step in our workflow, we leverage insights from earlier analyses to refine the model, enhancing the accuracy and practical applicability of findings. This strategic placement ensures that the model is robust and finely tuned to meet specific requirements.

Initially proposed by Young et al. [5], RSA involves categorizing input samples as ‘behavioral’ or ‘non-behavioral’ based on their alignment with expected model responses, this approach involves dividing samples by a threshold on the output space. Then, the input sets are compared to gain insight on the model behavior and mapping. The behavioral and non-behavioral outputs are then traced back to their originating sampled factors, where differences between the distributions of samples can be used to determine their significance in producing each part of the output, as shown in Eq. (7):

(7) $$S_{i}=\left|F_{x_{i}|y_{b}}\left(y\in Y_{b}\right)-F_{x_{i}|y_{nb}}\left(y\in Y_{nb}\right)\right|$$

where, $Y_{b}$ and $Y_{nb}$ represent behavioral and non-behavioral output sets, respectively, with $F_{x_{i}|y_{b}}$ and $F_{x_{i}|y_{nb}}$ denoting the ECDFs.

## 3 Application to pre-stressed modal analysis of penstock model

The dynamic issue of vibrations in penstocks, often exacerbated by hydraulic pulsations [67], heightens the risk of structural vulnerabilities, leading to a range of operational issues such as instabilities, material wear, thinning, and service interruptions [68]. A critical issue is the resonance phenomenon, which can induce substantial out-of-plane displacements, potentially culminating in fatigue damage to the penstock shell, even when vibration mitigative measures are implemented [7].

This section demonstrates the application of the systematic methodology described, integrating GSA with structural modal analysis to examine the free vibration characteristics of penstocks and advance our understanding of their dynamic behavior. This integration serves multiple purposes: it enhances model accuracy and reduces uncertainties by pinpointing critical parameters that significantly affect the system's response. Additionally, it guides future design and optimization efforts, thereby increasing the robustness and precision of investigations focused on the dynamic behavior and resonance phenomena in penstocks.

### 3.1 Model description

**Fig. 2:**
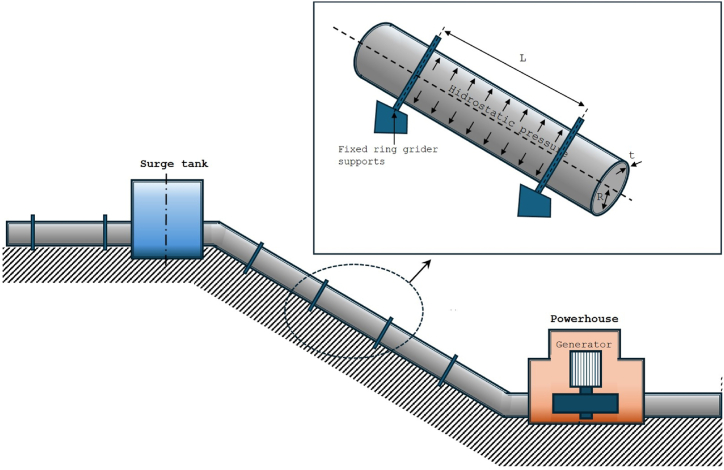
Shape of steel penstock analysis model.

The exposed penstock, typically a large-scale structure, was modeled using a single span as depicted in Fig. 2. To improve computational efficiency, a surface model focused on the mid-shell surface of the penstock was utilized. Due to the symmetrical nature of the geometry, loading, and boundary conditions along the yz-plane, only half of the span was modeled. The steel penstock's material considered is ASME SA-516 Grade 70, most popular pressure vessel grade of steel, in accordance with standard specifications for penstock structures. Initially, a numerical structural static analysis was conducted to ascertain the stress distribution within the penstock under internal hydrostatic pressure. This was followed by a free vibration analysis of the prestressed span to identify vibration frequencies and modes.

Adaptive mesh refinement was employed to ensure numerical precision, with convergence criteria set to a relative error of less than E = 0.01 %, as specified in Equation (8). The selected mesh size of 50 mm ensured that both results changes in static and modal analyses remained within the threshold upon additional refinement.

(8) $$Error=100\times \left(\frac{\phi_{i+1}-\phi_{i}}{\phi_{i}}\right)<E$$

where ϕ represents the result quantity, i denotes the refinement iteration, and E is the specified accuracy.

The penstock span model incorporated steady loads, accounting for hydrostatic pressure and gravity, which considered the weight of both the penstock and the contained water. Hydrostatic pressure was modeled to increase linearly with elevation, applying a typical pressure of 1.96 MPa for a 200m height span. Elastic supports simulated the ring girders’ constraining action and axial flexibility, for accurately capturing the structural behavior under load.

### 3.2 Free vibration characteristics

The derivation of natural frequencies for dynamic assessment typically relies on predefined loading conditions. However, the inherent variability in penstock excitation necessitates a comprehensive modal analysis first. Prioritizing modes based on participation factors and effective masses is essential for identifying the most critically influential natural frequencies and modes. These parameters are computed as shown in Eqs. (9), (10):

(9) $$\gamma_{i}=\phi i^{T}\left[M\right]D$$

(10) $$Meff,i=\gamma_{i}^{2}$$

where ϕ represents the mode shapes, [M] is the mass matrix, and D is the directional excitation vector. Participation factors (Eq. (9)) quantify the contribution of each mode to the overall dynamic response, while effective masses (Eq. (10)) measure the modal mass distribution. High values of these parameters highlight modes that are likely to be significantly excited under operational conditions, thus providing a focused approach for detailed structural evaluation.

The preliminary analysis involved extracting select natural frequencies and their respective vibration modes. By calculating mode participation factors, effective masses, and their respective ratios to total mass across six directional axes, the modes imparting considerable influence on the structure's dynamic response were discerned. Table 1 provides insights into the extent of effective mass engagement per mode. The summation of these ratios approaches unity, validating the capture of an encompassing and critical mode spectrum. The analysis required the selection of 3600 modes, achieving 90 % representation of the dynamic mass in three directions, and between 70 % and 80 % in the other three, capturing a substantial portion of the most influential dynamic behaviors.

**Table 1:** Modal analysis results. Modes with significant mass participation are highlighted in bold, highlighting their potential impact on structural behavior. The “Shape (n,k)” classification elucidates the mode shapes, with ‘n’ and ‘k’ indicating the number of circumferential and axial lobes, respectively.

Mode	Freq. (Hz)	Ratio eff. mass to total mass						Shape (n,k)
		X Dir.	Y Dir.	Z Dir.	X Rot.	Y Rot.	Z Rot.	
1	29,9355	**0,19,347**	1,09E-04	2,00E-15	7,96E-05	**0,141,273**	4,06E-05	(2, 1)
2	31,5997	3,09E-05	**0,69,294**	1,84E-11	**0,50,598**	2,24E-05	**0,27,722**	(1, 1)
3	40,8313	4,16E-13	8,91E-09	4,86E-11	2,48E-09	**0,03144**	4,28E-09	(2, 2)
4	51,4747	2,51E-06	6,60E-12	1,64E-12	4,88E-12	1,73E-06	5,97E-03	(3, 1)
5	53,4899	1,75E-08	6,50E-14	2,26E-14	1,49E-11	8,39E-07	2,04E-12	(3, 2)
6	58,9733	8,17E-06	1,63E-13	4,68E-11	2,56E-13	5,66E-06	1,17E-03	(3, 3)
7	61,7522	**0,03963**	9,98E-10	8,05E-14	5,95E-09	**0,02894**	7,04E-08	(2, 3)
8	69,0612	6,21E-08	2,00E-13	2,74E-13	2,63E-10	1,51E-07	1,40E-14	(3, 4)
9	73,8403	6,57E-10	1,26E-12	4,26E-08	**0,12,226**	1,94E-08	7,53E-13	(1, 2)
10	77,7084	9,23E-03	1,12E-14	4,68E-15	4,54E-09	6,74E-03	4,44E-08	(4, 1)
…	…	…	…	…	…	…	…	…
30	124,516	9,48E-08	**0,13,972**	6,34E-08	**0,10,203**	7,14E-08	**0,05426**	(1, 3)
32	126,034	0,00E+00	1,48E-08	7,78E-01	6,71E-09	1,62E-03	7,22E-09	(0, 2)
102	234,731	5,01E-09	**0,04755**	5,59E-11	**0,03472**	1,46E-08	1,69E-02	(1, 5)
220	371,522	7,88E-14	1,48E-10	**0,07989**	7,17E-12	1,49E-04	1,48E-11	(0, 4)
704	694,931	**0,14,365**	7,15E-07	6,75E-08	3,01E-06	**0,1042**	9,41E-09	(0, k)
709	696,835	**0,19,731**	1,14E-06	1,07E-05	5,60E-07	**0,15,791**	3,98E-07	(0, k)
…	…	…	…	…	…	…	…	…
3598	2716,71	8,68E-08	7,48E-09	2,57E-09	2,43E-06	2,30E-07	2,63E-09	–
3599	2717,83	1,89E-07	5,25E-07	2,72E-09	3,93E-06	3,41E-07	4,99E-07	–
3600	2718,72	1,84E-07	2,94E-06	1,90E-11	3,38E-06	7,48E-07	2,99E-06	–
sum	–	8,04E-01	9,28E-01	9,37E-01	8,96E-01	7,77E-01	7,23E-01	–

The mode exhibiting the highest participation factor in a given direction is deemed most consequential, indicating a significant portion of the structural mass actively partaking in that specific mode's vibration. Table 1 delineates the individual contributions of each mode across various directions. Notably, the mode of order 709 (with a frequency of 696.8 Hz) is the most significant in the X direction, followed by the fundamental mode (with a frequency of 29.9 Hz), which contributes to 19.3 % of the mass displacement.

In the Y direction, modes 2 and 30 contribute significantly, with 69.3 % and 14.0 % of the mass movement, respectively. Similarly, mode 220 stands out in the Z direction with a 79 % contribution. Prominent modes in the rotational directions (X, Y, and Z) are also identified, with significant mass displacements linked to a select few modes, as detailed in the table. Notably, modes 1 and 2 exhibit high ratios across multiple directions, underscoring their potential for excitation and necessitating further scrutiny. At least 70 % of the moving mass in each direction is attributed to a few notable modes, emphasizing their significance and the necessity for focused attention. It is worth noting that pertinent to consider even minimally contributing modes to the dynamic response, adhering to industry standards that caution the inclusion of modes with over a 1 % contribution. High-frequency modes warrant attention for their potential noise implications, a notable concern in penstocks [[69], [70], [71]].

Table 2 compiles the initial five mode shapes, along with modes 30, 32, and 102, which are the last most pronounced mode shapes within the frequency threshold of 300 Hz. Areas of maximum deformation for each mode are highlighted in red, providing visual cues for understanding the deformation patterns.

**Table 2:** Overview of key mode shapes across the span, showing deformation patterns by circumferential (n) and axial (k) lobes, within low frequency range (<300 Hz).

Mode Order	Natural Freq. (Hz)	Mode type (n, k)	Mode shape
1	29.9355	(2, 1)	
2	31.5997	(1, 1)	
3	40.8313	(2, 2)	
4	51.4747	(3, 1)	
5	53.4899	(3, 2)	
30	58.9733	(1, 3)	
32	61.7522	(0, 2)	
102	234.731	(1, 5)	

The fundamental breathing mode at 29.93 Hz (n = 2, k = 1) exhibits the greatest deformations at the lobe centers. The second-order bending mode at 31.59 Hz behaves akin to a beam, showcasing maximum deformation centrally and minimal near supports. This description extends to various modes, outlining their deformation patterns and frequencies.

Bending mode shapes signified by (n = 1) with odd ‘k’ values, are predominantly excited in the Y direction, influencing vertical dynamics. Lateral and rotational dynamics are characterized by breathing modes with ‘n = 2′ and odd ‘k’ values, confirming their significance across different directions of movement.

These insights inform the vibrational tendencies of penstocks, aiding in the prediction and mitigation of potential issues due to vibrations. It is important to note that this analysis is deterministic and the conclusions presented are model-specific. They do not allow for generalizations about critical modes or natural frequencies to avoid, as the results may be influenced by various changes. Therefore, probabilistic analysis is required to verify if these results remain valid and to reduce uncertainty.

### 3.3 Uncertainty analysis

Performing UA beforehand furnishes the experimental groundwork for SA. A pivotal initial step in data analysis or modeling endeavor involves comprehensively understanding the distribution of mode shapes and their frequencies. Questions such as the span of frequencies observed, the frequency ranges predominantly associated with each mode of interest, their central tendencies, and the presence of skewness or bimodality are essential. Additionally, evaluating the probability proportions linked to different vibrational frequency ranges and discerning the nature of correlations or trends between input parameters and natural frequencies are crucial for analyzing structural vibrational behavior.

These inquiries are addressed in this section through UA as following: (i) Propagating uncertainties to gauge the span of potential vibrational frequencies a penstock span might encounter (Sect. *3.3.1*), (ii) Visualizing frequency distributions to ascertain variations and define vibrational limits (Sect. *3.3.2*), (iii) Utilizing ECDFs to determine frequency range probabilities and the likelihood of surpassing critical frequency thresholds, facilitating an implicit reliability assessment (Sect. *3.3.3*), and (iv) Exploring input-output relationships through scatterplots to discern trends and select the most suitable SA methods for further investigation (Sect. *3.3.4*).

#### 3.3.1 Uncertainty propagation and GSA setup

Addressing the uncertainty in model responses involves propagating the uncertainty within the deterministic structural model of the penstock. This step is crucial for setting up the subsequent GSA. The initial phase involves the meticulous selection of model outputs and the parameters contributing to uncertainties in these outputs, along with the definition of input variability, including their ranges and their PDFs.

While sensitivity analysis often considers multiple outputs collectively for a comprehensive view, our approach examines each output independently, acknowledging that different mode shapes may exhibit unique sensitivities. This allows for detailed scrutiny of individual sensitivities, providing profound insights into penstock structural behaviors. This specific application focuses on nine structural factors as sources of uncertainty (Table 3), based on their critical impact on the dynamic behavior of penstocks for several key reasons. Uncertainties related to geometric properties such as penstock radius, wall thickness, and span length are considered fundamental, as they directly influence the natural vibration modes and resonance frequencies, which are crucial for structural safety and design. These parameters are subject to variability due to manufacturing tolerances, wall thinning and fatigue damage, and deformations induced by pressure pulsations, justifying their inclusion in the uncertainty analysis. Uncertainties related to stiffening elements, such as circumferential stiffeners and their configuration, have been incorporated due to their potential impact on the overall structural rigidity. Although these parameters may appear less influential individually, they interact in complex ways with other geometric parameters, leading to significant non-linear effects on the vibrational response. Finally, variations in the hydraulic head directly influence the internal pressure, which, in turn, significantly affects the penstock vibrational responses, making it a critical source of uncertainty for this specific application.

**Table 3:** Uncertain parameters considered for the penstock span.

Variable	Description	Distribution
t	shell thickness	Uniform (12–42 mm)
L	Span length	Uniform (5–30 m)
R	Radius	Uniform (500–2500 mm)
H	Piezometric head of span	Uniform (0–1500 m)
shape	stiffener shape	I and T shape
$n_{s}$	stiffeners per span	1,2,3
${\;h}_{s}$	stiffener length (of flange and web)	Uniform (50–200 mm)
$t_{s}$	thickness of stiffener (of flange and web)	Uniform (50–200 mm)

In the input range selection, given the absence of observational data, and guided by literature (e.g., Nakamura et al. [7]), we employ a uniform PDF for each parameter through the LHS method to efficiently capture parameter impacts and support multiple GSA methods, thereby optimizing computational resources. A carefully chosen sample size of 1000 evaluations, based on LHS literature and convergence testing, ensures a balance between computational efficiency and analytical precision.

Evaluations, conducted via parametric modeling [72] (in Ansys Workbench's DesignXplorer), enable a systematic exploration and iteration of design variations thereby propagating uncertainties in the free dynamic penstock model.

**Fig. 3:**
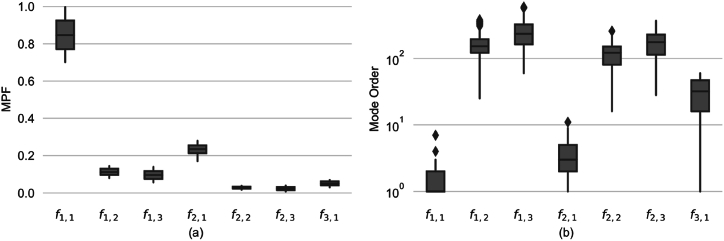
(a) Distribution of Mode Participation Factors for seven distinct structural mode shapes, indicative of their contribution to overall vibration behavior, and (b) The range of their mode orders, highlighting the spread and central tendencies (on a logarithmic scale).

The analysis identified fundamental and prominent frequencies, pinpointing modes with Mode Participation Factors (MPFs) surpassing 2 %. Fig. 3 a depicts the MPF distributions for these noteworthy modes, whereas Fig. 3 b displays the distribution of their frequency orders. Notable findings are as follows.

• The shape of significant modes remains consistent across evaluations, suggesting that the factors considered primarily affect the frequency and order of the modes, rather than altering their shapes.
• Frequencies $f_{2,1}$ and $f_{1,1}$ emerge as particularly noteworthy, boasting the highest MPFs and advanced orders simultaneously, distinguishing them from other modes with relatively low MPFs.
• Despite its relatively low MPF of below 6 %, mode $f_{3,1}$ spans a broad ranking interval, including early ranks. This suggests its potential significance when it appears as a fundamental frequency or early in the frequency sequence. In contrast, modes $f_{1,2}$, $f_{1,3}$, $f_{2,2}$, consistently appear in later ranks with low MPFs, making them less likely to be activated and contributing insignificantly to the system's total vibrational energy.

Therefore, based on the analysis, the modes $f_{1,1}$, $f_{2,1}$, and $f_{3,1}$, corresponding to the bending natural frequency and the oval modes with two and three circumferential lobes, respectively, have been identified as the most significant and are selected for further investigation in this GSA study.

#### 3.3.2 Natural frequency distributions and probabilities

**Fig. 4:**
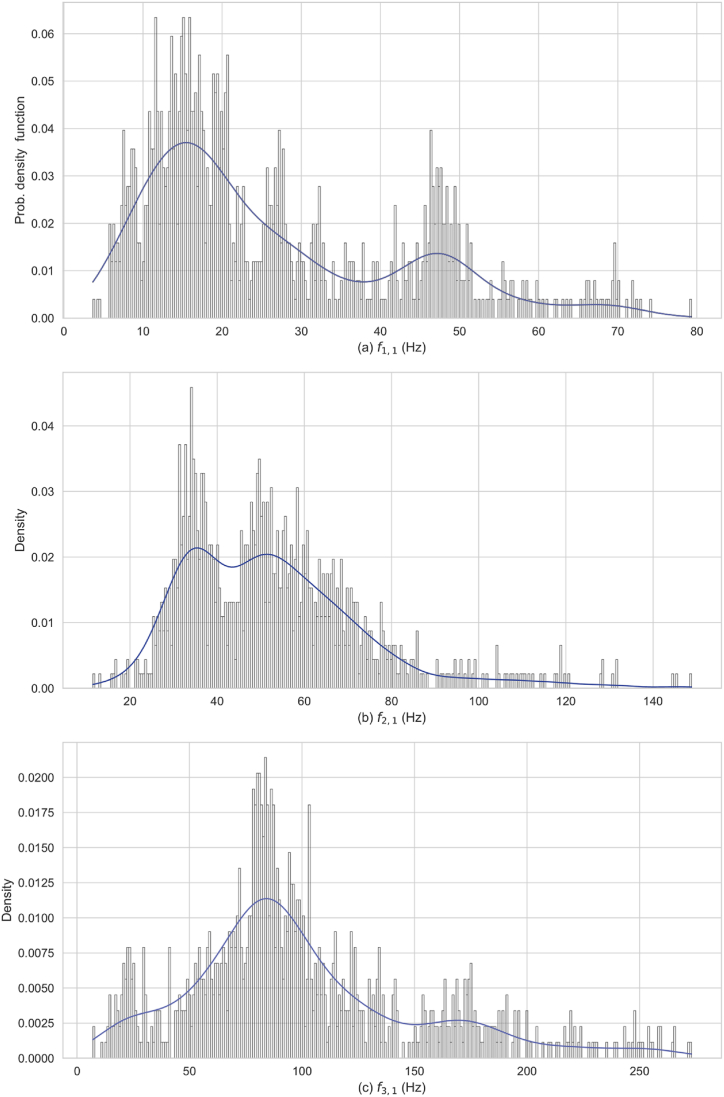
Histograms with overlaid KDE representing the distributions of mode shapes a) $f_{1,1}$, b) $f_{2,1}$, and c) $f_{3,1}$. These plots depict the frequency densities and illustrate the diversity and concentration of occurrences within the spectrum.

This section presents the density distribution analysis of the three prominent natural frequencies $f_{1,1}$, $f_{2,1}$, and $f_{3,1}$ derived from uniform input distributions by LHS design. The analysis incorporates the uncertain parameters detailed in Table 3. Fig. 4 displays histograms that approximate the PDFs by segmenting the natural frequency axes into 300 bins, with density normalization ensuring the summed area of the bars equals one. The PDFs are used to visualize the distribution and variability in the dynamic behavior of the penstock structure, and to highlight regions with concentrated values, with specific frequencies more likely to dominate the specific mode under analysis. To mitigate the influence of discrete bin selection on the appearance of the distribution, Kernel Density Estimation (KDE) overlays the histograms, by providing a Gaussian-smoothed continuous density estimate, thus capturing data trends more accurately.

• From the PDF of $f_{1,1}$ (Fig. 4a), the histogram covers a frequency range from approximately 4 to 80 Hz. Notable peaks are observed between 10 and 20 Hz, with additional smaller but significant peaks at approximately 7.5 Hz, 27 Hz, and 48 Hz. The presence of these peaks indicates multiple critical frequencies resulting in this bending mode of the penstock. These peaks indicate likely predominant bending frequency values. The KDE curve illustrates a bimodal distribution.
• The density distribution of the two-lobed oval mode $f_{2,1}$ (Fig. 4b) covers a frequency range of from approximately 8 to 140 Hz. The histogram indicates prominent peaks and concentrations around 34 Hz and 50 Hz, suggesting that this frequency range in $f_{2,1}$ is critical. The KDE overlay illustrates an asymmetrical and bimodal distribution with a right skew indicating a complex pattern.
• The density distribution of the three-lobed oval mode $f_{3,1}$ (Fig. 4c) extends to 250 Hz, indicating a broad dispersion of frequency values. The primary peak is around 80–85 Hz, with most frequencies concentrated between 50 and 100 Hz. Smaller peaks are scattered throughout the frequency range, suggesting indicate additional contributing frequencies, albeit to a lesser extent. The right skewness and broad dispersion of the $f_{3,1}$ frequency distribution indicate a complex pattern, deviating from a normal distribution. The distribution tails off towards higher frequencies, suggesting they are less likely to dominate the structure's dynamic response.

A broad frequency response is observed in the oval modes $f_{2,1}$ and $f_{3,1}$, displaying greater frequency dispersion compared to the bending mode $f_{1,1}$. This wider spread may indicate heightened sensitivity of these modes to variations in the penstock's structural parameters. The observed multimodal and non-normal distributions suggest nonlinear behaviors, likely due to complex interactions between structural parameters. These findings imply that the structural dynamics of the penstock could be influenced by a diverse range of excitations. The intricate patterns in the frequency distributions emphasize the need for further investigation and sensitivity analysis to uncover the underlying factors driving these distributions and to assess their implications on vibration modes.

#### 3.3.3 Resonance risk assessment via ECDFs

**Fig. 5:**
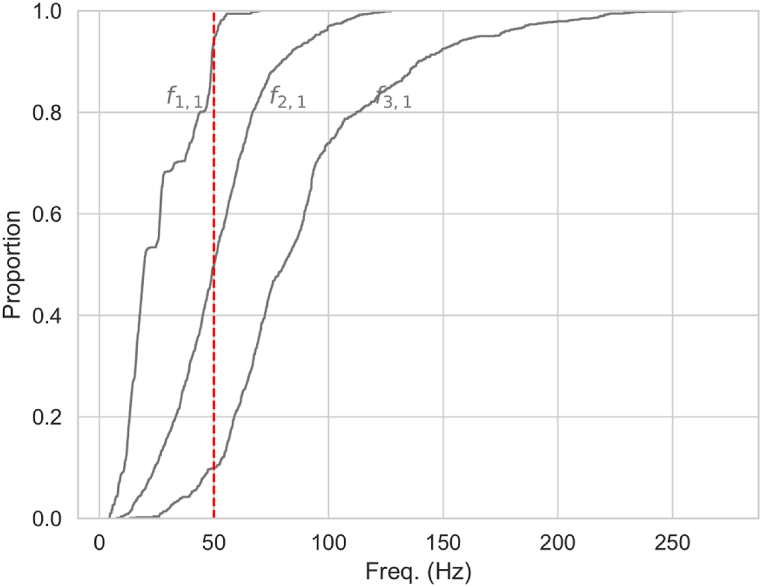
Empirical Cumulative Distribution Function of mode shapes $f_{1,1}$, $f_{2,1}$, $f_{3,1}$, demonstrating the probability of resonance occurrence relative to a critical 50 Hz frequency threshold.

The ECDFs, as presented in Fig. 5, serves as a crucial visual tool for assessing resonance risks. It enables the estimation of probabilities that natural frequencies will fall within specified thresholds, thereby informing strategies for vibration control.

External vibrations impacting the penstock include hydraulic pressure fluctuations, as detailed in Ref. [67]. These are primarily driven by the specifications of hydraulic turbines as follows: (i) Hydraulic turbine rotation speed N (rpm) yields a frequency $f_{w}=N/60$; (ii) Blade count Z per runner $f_{w}=NZ/60$; (iii) Water vortex frequency in the draft tube $f_{w}=N/\left(60\times 3.6\right)$.

For instance, Francis turbines operating between 500 and 2500 rpm, with a blade count ranging from 9 to 19, generate rotational frequencies extending from approximately 8.33 Hz–41.66 Hz. Additionally, the frequency of the vortex in the draft tube varies between 2.31 Hz and 11.6 Hz. Considering the interaction with the number of blades, the induced frequency can range from 75 Hz to 792 Hz. Taking into account the resonance zone, defined as ±20 % of the specified vibration, as indicated in the ASCE design guide for penstocks, these calculations suggest that the 50 Hz threshold marks a critical frequency. Frequencies below this threshold may indicate risks of vibrational issues. The analysis of the ECDFs curves for the vibrational modes considered further elucidates the potential risks associated with these frequencies, as shown in Fig. 5.

• The ECDFs curve of $f_{1,1}$ ascends sharply from the start, indicating that most bending frequency values fall below 50 Hz, with nearly 92 % of $f_{1,1}$ values under this critical threshold, signifying prevalent low-frequency bending. Additionally, there is a 10 % probability that $f_{1,1}$ values fall between 45 and 50 Hz.
• The ECDFs curve of $f_{2,1}$ curve rises less steeply compared to $f_{1,1}$ showing a broader dispersion of $f_{2,1}$ values, with a proportion of 38 % below 50 Hz and approximately 25 % falling within the 45–55 Hz range.
• Demonstrating even greater dispersion than $f_{2,1}$, only 18 % of $f_{3,1}$ values fall below 50 Hz, indicating a propensity for higher frequency occurrences.

From these observations, to effectively dampen vibrations at the critical 50 Hz threshold (resulted from turbine), efforts should be primarly directed toward managing the oval mode $f_{2,1}$. The bending mode $f_{1,1}$ is most likely to manifest in lower frequencies whereas $f_{3,1}$ showing potential to occur at higher frequencies posing less of a risk in the 50 Hz range.

#### 3.3.4 Preliminary sensitivity assessment

The scatter plots depicted in Figs. A1. *1*, ***A.1.2***, and ***A.1.3*** (Appendix A.1): elucidate the relationships between parameter variations and the natural frequencies of the mode shapes $f_{1,1}$, $f_{2,1}$, and $f_{3,1}$, respectively. Observations from Scatterplot Analysis:

• Bending mode $f_{1,1}$ (Figure A1. *1*): Clear influences from radius (R) and length (L) variations are visible. A larger radius narrows the frequency range, while increased length inversely affects $f_{1,1}$ frequencies. This aligns with mechanical expectations, underscoring the significant roles of radius and length in penstock bending dynamics. Conversely, thickness, slope, head, and stiffener parameters show dispersed relationships with natural frequencies, suggesting complex interactions and potentially nonlinear behaviors not immediately apparent from scatter plots. The Point plot $f_{1,1}$ illustrate the effect of adding circumferential. Both I-shape and T-shape stiffeners increase $f_{1,1}$, with significant jumps observed when transitioning from one to two uniformly distributed stiffeners, enhancing structural rigidity. The initial addition of a central stiffener has a less pronounced effect, attributed to mass distribution impacts. Error bars indicate consistent precision across measurements for I-shape, while uncertainty decreases with more stiffeners for T-shape, suggesting stiffener number impacts bending frequency more than shape.
• Oval mode $f_{2,1}$ (Figure A1. *2*): Trends are noted in radius, section length, and internal pressure changes, with each affecting the natural frequencies. Increases in radius and length lead to frequency decreases, while Internal pressure correlates positively with frequency increases. Other parameters show no clear trend. The Point plots $f_{2,1}$ reveal that stiffener additions elevate the second natural frequency, especially noticeable with T-shaped stiffeners at the third addition, highlighting stiffener quantity and arrangement's critical role in frequency enhancement.
• Oval mode $f_{3,1}$ (Figure A1. *3*): The trends are similar to $f_{2,1}$ but with an upward frequency shift, indicating a baseline structural response change with stiffener additions, yet following similar increment patterns.

While direct influences of some parameters on frequencies are evident, others present complex, indirect roles due to nonlinear interactions requiring advanced analysis to dissect their contributions.

### 3.4 Factor screening for model reduction

**Fig. 6:**
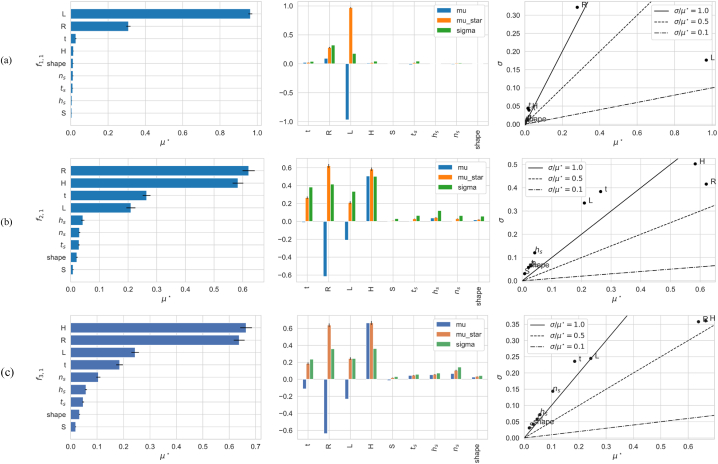
Morris sensitivity measures (μ, μ∗, and σ) for output responses: a) $f_{1,1}$, b) $f_{2,1}$, c) $f_{3,1}$,1, to the nine parameters in Table 3. The left panel shows the mean absolute values of the elementary effects (EEs) for ranking. The middle column displays bar charts with μ (direction of effect), μ∗ (mean absolute effect), and σ (standard deviation of EEs). The right column presents covariance plots correlating μ∗ and σ, with reference lines indicating linear, nonlinear, or interactive influences.

This analysis employs the extended version of the Morris method for factor screening, to identify non-influential parameters that can be fixed within their uncertainty ranges without significantly altering model outcomes, thereby discarded from subsequent analysis. By varying the nine factors across ten levels and using a sampling size of N = 600 and optimal trajectories [37,73,74] of T = 60, the method conducts T×(k+1)=600 model evaluations. Bootstrapping verification with 100 iterations ensures the robustness of the screening results, achieving an optimal balance between accuracy and computational efficiency. The results obtained for the Morris screening analysis with confidence interval level of 0.95 are displayed in Fig. 6 which include:

• **Screening analysis for the bending mode shape**$f_{1,1}$ (Fig. 6a): The span length showcases the highest mean negative effect μ but also the highest mean absolute effect $\mu^{*}$ signaling its predominant influence on reducing $f_{1,1}$, indicative of a linear relationship. Its scatter plot positioning in the $\mu^{*}$ ≫ σ zone suggests a consistent effect, almost independent of other parameters. In contrast, the penstock radius R falls into a zone where $\mu^{*}$ < σ, denoting variability in effects due to interactions or curvature influences, yet with a notable positive impact reflected by a high $\mu^{*}$. The Stiffener parameters $t_{s}$, $n_{s}$, and $h_{s}$, alongside their shape, though impacting less directly, their significant σ values highlight a variability suggesting more pronounced effects in interaction than in isolation. The inclination S of the penstock exhibits minimal influence on $f_{1,1}$, as denoted by its negligible $\mu^{*}$ and σ values, indicating a potential area for model simplification by fixing this parameter without affecting model accuracy significantly.
• **Screening analysis for oval mode shapes**$f_{2,1}$ (Fig. 6b) and $f_{3,1}$ (Fig. 6c): Radius R and head H emerges as a critical parameter for both $f_{2,1}$ and $f_{3,1}$, with high $\mu^{*}$ showing almost similar values and significant influence. However, R has negative mean effect μ, indicating that higher values correlate with lower frequencies in the oval modes' shapes. Length and thickness also show considerable $\mu^{*}$ and positionne at $\mu^{*}$ < σ indicating significant and variable effects. Parametres of Stiffeners show limited sensitivity towards oval modes, evidenced by their relatively low $\mu^{*}$ with σ slightly superior, indicating some nonlinear interactions with other variables.

Across all frequencies, slope demonstrates negligible effects, underscoring its limited influence within the studied range.

The Screening analysis reveals complex parameter interactions with distinct impacts on vibrational modes. Length and radius are key for bending frequencies, while radius and head dominate oval frequency sensitivities. The slope parameter, showing minimal sensitivity, was discarded from subsequent analysis without compromising predictive accuracy. Regarding the stiffener parameters, although their mean effects were lower, their roles are potentially more nuanced and complex.

Stiffener parameters (shape, height, and thickness) were aggregated into a single representative parameter, the moment of inertia ratio I / I 0. Here, I 0 represents the moment of inertia for shell wall calculated using the effective width of $1.56*\sqrt{r\cdot t}$ [67], and I is the moment of inertia for shell wall in addition to circumferential stiffener. This ratio encapsulates the combined effect of these parameters on structural rigidity. This simplification preserves the integrity of their interactions while reducing model complexity. Consequently, the model was streamlined from nine to six parameters, optimizing practicality and allowing for a more focused investigation of the most influential parameters, thereby enhancing the model's efficiency and relevance.

### 3.5 Global sensitivity analysis

This section aims to prioritize the factors influencing mode shapes by employing a variety of GSA techniques. The techniques used include EFAST and RBD-FAST for variance-based analysis, and DMIM and PAWN for density-based sensitivity analysis, allowing for comparative evaluation (cf. Sect. *2.3* for criteria for selecting these methods). An overview of the employed techniques and their parameter setup is provided in Table 4. All sensitivity analyses were executed using the SALib library [75,76].

**Table 4:** Experimental set-up for GSA methods used in this study.

Method	Approach/setting	Sampling Method	Method Parameters	Cost of analysis
Extended Morris [38,39]	Elementary Effects/FF	OAT sampling	k = 9; N = 600; grid levels = 10; optimal traject. T = 60	T×(k+1)
EFAST (total-order index) [59]	Variance/FP	FAST sampling	$M_{s}$^1^ = 4; N = 500; k = 6	k×N
RBD-FAST (first-order index) [57,58]	Variance/FP	LHS	N = 1000; ${\;M}_{s}$ = 10	N
PAWN^2^ [64,65]	Density/FP	LHS	conditioning intervals S = 10	N
DMIM [62,63]	Density/FP	LHS	N	N
Sobol’ first-order index [48]	Variance/FP	LHS	N	N
RSA^3^ [66]	Density/FM	LHS	N; bins = 20	N

k denotes the number of factors; N the base sample size. The cost of analysis corresponds to the required number of runs. The number of resample used to compute the confidence intervals equals 100 and confidence interval level equals 0.95.^1^Ms is the number of harmonics considered in the Fourier series decomposition. ^2^ Kolmogorov-Smirnov (KS) test is used to compare distributions. ^3^The two-sample Cramér-von Mises (CvM) test is used to compare distributions.

**Fig. 7:**
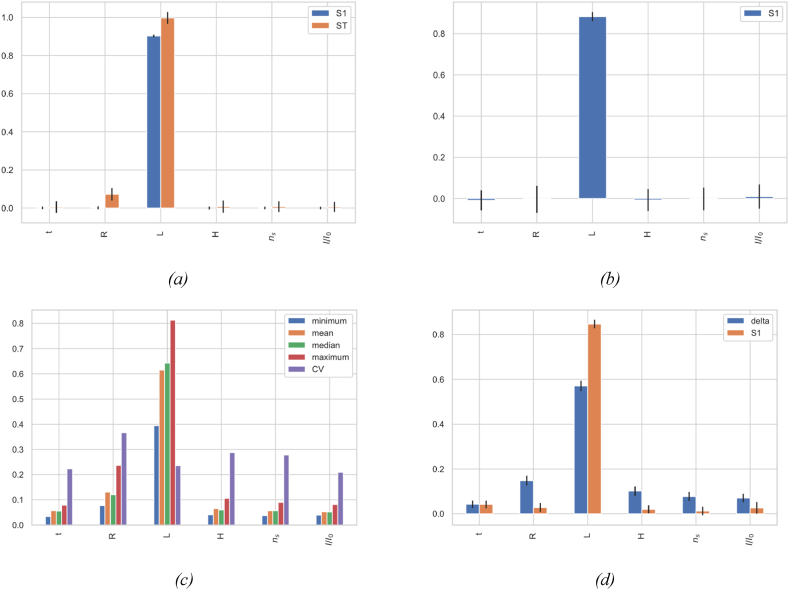
Global sensitivity analysis results and comparative evaluation of GSA techniques for the bending mode shape $f_{1,1}$: (a) EFAST: first-order (S1) and total-order (ST) indices. (b) RBD-RSA: first-order index (S1). (c) PAWN: statistics including min, mean, median, max (using Anderson-Darling for ECDFs divergence), and coefficient of variation (CV). (d) DMIM: Delta index and Sobol’ first-order index (S1).

**Fig. 8:**
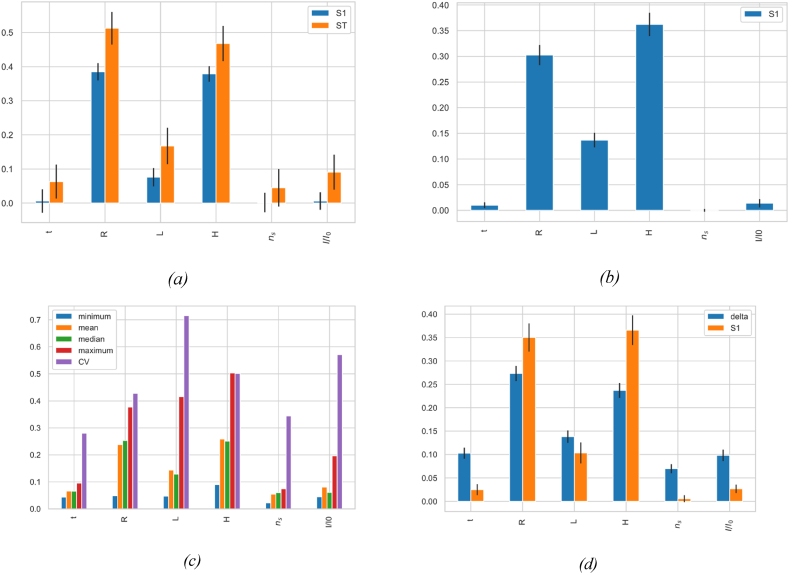
Global sensitivity analysis results and comparative evaluation of GSA techniques for the oval mode shape $f_{2,1}$: (a) EFAST, (b) RBD-RSA, (c) PAWN, (d) DMIM.

The DMIM analysis in Fig. 7d, *8*d, and *9*d is complemented by the Sobol’ first-order index S1, which shows the direct effects of parameters. While DMIM displays overall effects, including both direct and interaction, S1 isolates the direct contributions of each parameter, providing detailed understanding of their individual impacts.

**Fig. 9:**
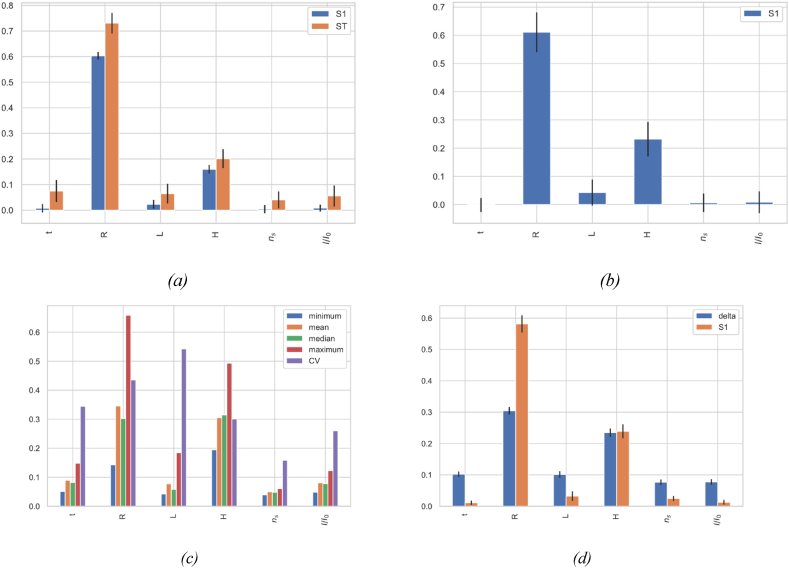
Global sensitivity analysis results and comparative evaluation of GSA techniques for the oval mode shape $f_{3,1}$: (a) EFAST, (b) RBD-RSA, (c) PAWN, (d) DMIM.

• **Methodological Robustness**: The use of various GSA methods inherently contributes to the robustness and reliability of the results. Convergence studies for all analyses were conducted to evaluate the impact of sample size on sensitivity indices, indicating that sample sizes indicated in Table 4 are necessary for precise results. Similar tests for the methods parameters, which was adjusted for each technique to maintain the stability of the factor influence hierarchy. Further robustness checks were performed through bootstrapping without replacement across all methods, involving 100 bootstrap resamples. This process calculated the averages and confidence intervals for each sensitivity index, ensuring robust and reliable analysis.
• **Sensitivity of the bending mode**$f_{1,1}$: Fig. 7 reveals clear trends in the influence of the various structural parameters. Span length L stands out as the most influential, exhibiting notably high sensitivity indices across all the four analysis methods (Fig. 7a–d). This predominance aligns with the principles of structural dynamics, where beam length is a key determinant of natural bending frequencies in extended structures. In contrast, secondary parameters such as span radius R, head H, number of stiffeners $n_{s}$, and the relative stiffness of stiffeners I / I 0 generally exhibit lesser influence. However, their impacts vary depending on the sensitivity measure employed. The PAWN method (Fig. 7c), which analyzes ECDFs, reveals significant variability in the contribution of these parameters, indicating complex interdependencies among them. Furthermore, the delta measure from the DMIM method (Fig. 7d) corroborates these interactions, underscoring the necessity of evaluating the entire response distribution to accurately assess the sensitivity of the bending modes in penstock structural behavior.
• **Sensitivity of oval mode shape**$f_{2,1}$**:** The GSA results showed in Fig. 8a–d delineates a clear distinction between variance-based and density-based methods. Variance-based methods identify radius R and head H as predominant factors, as evidenced by high first-indices (S1) in EFAST (Fig. 8a) and RBD-FAST (Fig. 8b), and by their significant contributions to total variance in EFAST (ST). Density-based methods (Fig. 8c and d), while corroborating these findings, also reveal high variability in the impacts of span length L through the high coefficient of variation (CV) in Fig. 8 c, suggesting a non linear sensitivity. Also, these methods expose broader effects of wall thickness t and relative stiffener stiffness I / I 0, capturing nuanced interactions, overlooked by variance-based approaches.
• **Sensitivity of oval mode shape**$f_{3,1}$**:** From Fig. 9a–d, both variance-based and density-

Based methods confirm the significant influence of span radius R, as demonstrated by exceptionally high first indices in EFAST (Fig. 9a) and RBD-FAST (Fig. 9b), and a high coefficient of variation in the PAWN method (Fig. 9c). Notably, the DMIM and PAWN methods (Fig. 9d and c, respectively) reveal that H exhibits considerable variability in its influence. While span length L, the number of stiffeners $n_{s}$, and the relative stiffness of stiffeners I/I0 undoubtedly affect the dynamic response, they are less critical than R and P in influencing the $f_{3,1}$ mode shape.

### 3.6 Regional sensitivity analysis

RSA does not require a tailored sampling strategy, allowing it to be applied without incurring additional computational expense. Modelers can utilize any design drawn from previous methods used in the workflow; in this case, we employed the previously used LHS. RSA can be implemented in various ways, each providing distinct insights into the model's behavior according to the targeted objectives.

#### 3.6.1 ECDFs analysis

**Fig. 10:**
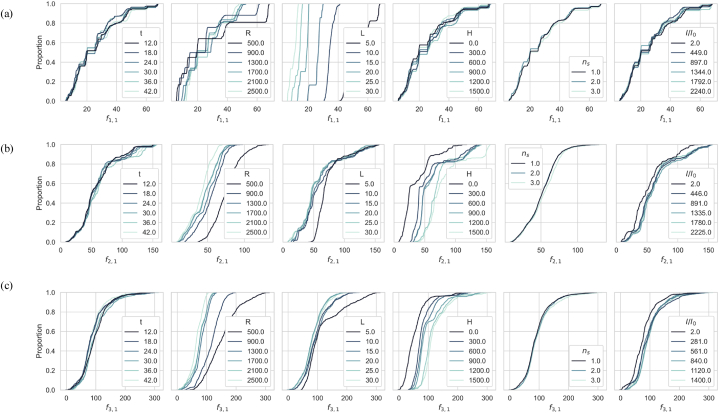
ECDFs curves used for RSA, visually mapping the sensitivity of mode shapes: (a) $f_{1,1}$, (b) $f_{2,1}$, and (c) $f_{3,1}$.

The ECDFs curves, previously used to calculate sensitivity estimates for PAWN method, enable a qualitative assessment and cost-effective approach for RSA by visually mapping the parameters and mode shapes sensitivity. The observations from Fig. 10 are as follows.

• The ECDFs curves for the bending mode $f_{1,1}$ (Fig. 10a) exhibit a pronounced separation for span length L, confirming the significant influence. Higher L values shift the distribution leftward, signifying lower frequencies, correlating increased length with decreased bending mode frequencies. Similarly, span radius R shows notable separation in ECDFs curves, indicating high sensitivity to $f_{1,1}$; Higher R values tend to reduce the spread of the $f_{1,1}$ distribution, signifying more concentrated frequencies. Conversely, lower R values broaden the distribution, indicating greater variability in the bending mode frequencies $f_{1,1}$. In contrast, the thickness t shows clustered ECDFs curves, indicating relatively low influence, with lower values shifting the distribution slightly leftward, suggesting lower bending frequencies. I / I 0 shows relatively moderate separation, with higher I / I 0 values shift the distribution rightward, indicating higher frequencies. Lastly the number of stiffeners $n_{s}$ shows minimal separation, indicating relatively low influence on $f_{1,1}$. Different $n_{s}$ values do not significantly affect the distribution, suggesting low sensitivity.
• The ECDFs curves for $f_{2,1}$ response (Fig. 10b) exhibit pronounced separation, indicating high sensitivity; higher R values reduce the spread, concentrating bending frequencies, while lower R values increase variability. The thickness t and $I/I_{0}$ shows closely clustered curves, indicating relatively low influence, with lower values slightly shifting the distribution leftward, suggesting lower bending frequencies. The span length shows separation in ECDFs curves, indicating moderate sensitivity to $f_{1,1}$; higher L values broaden the distribution, suggesting a wider range of frequencies, while lower L values narrow it. The head H shows clear separation, indicating high sensitivity, with higher H values shifting the distribution rightward, indicating higher frequencies. Lastly, the number of stiffeners $n_{s}$ shows minimal separation, indicating the least influence.
• Fig. 10c displays the ECDFs curves for the oval mode shape $f_{3,1}$ which exhibit similar trends to those observed for $f_{2,1}$; R and H remain the most influential parameters, L shows moderate sensitivity, and t, $n_{s}$, and $I/I_{0}$ have the least sensitivity, similar to $f_{1,1}$. The key difference from $f_{2,1}$ is that the range of bending frequencies for $f_{3,1}$ is broader, suggesting a greater variability in the mode shape.

#### 3.6.2 Monte Carlo Filtering

In our approach, we initially chose broad parameter ranges to capture any potential impacts on model outcomes. However, this inclusivity might introduce poorly performing parameter values, impacting the SA's effectiveness. To address this, the Monte Carlo Filtering (MCF) [15] is applied for focused sensitivity analysis.

Applying a targeted performance filter streamlines the SA by focusing parameters critical to specific behaviors. This technique refines and revises the model setup for a more focused analysis. It also aligns the sensitivity assessments with practical engineering objectives, enhancing the relevance of solutions to penstock design.

The MCF method combines GSA with a performance-based screening of simulation outcomes. This method aims to exclude poorly performing simulations that could bias sensitivity index estimations. A performance-based screening identifies ‘behavioral’ from ‘non-behavioral’ simulations; those producing responses within and out of a defined threshold (50hz in this case), respectively.

**Fig. 11:**
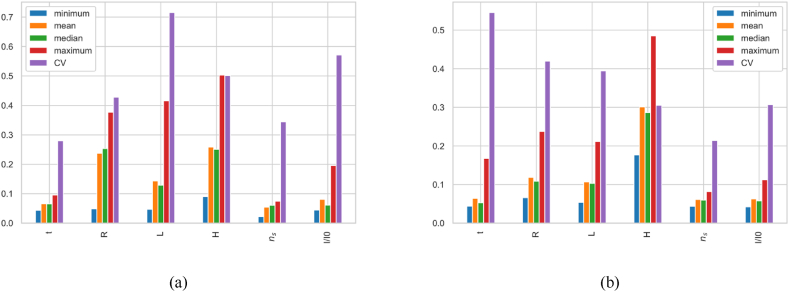
Monte Carlo Filtering using PAWN method: (a) Results without filtering, (b) Results with 50 Hz threshold filtering.

Fig. 11 contrasts the PAWN sensitivity indices computed with all simulations (Fig. 11a) against those recalculated after excluding poorly performing simulations (Fig. 11b), utilizing the MCF analysis with the 50 Hz filtering threshold. The results are that this filtering significantly alters the parameter influence ranking on $f_{2,1}$, shifting the radius and head rankings. This underscores how performance-based filtering refines SA results.

The adjustment reveals that the sensitivity of these structural factors varies across the mode frequency sub-ranges of interest. Removing the poorly performing simulations changed the ranking of parameter influence on $f_{2,1}$. Considering the entire frequency range (Fig. 11a), the radius and head are equally the most influential parameters, followed by the length. However, when focusing on the subrange (⟨< 50 Hz) (Fig. 11b), the radius becomes less influential and holds the same importance as the length, while the other parameters maintain their positions.

#### 3.6.3 Segmented response space analysis

For refining understanding of parameter sensitivities, we applied an extension of the MCF approach, segmenting the response space into equal output sample groups for a nuanced evaluation of input sensitivities. The distributions of behavioral (within target region) versus non-behavioral (outside target region) outputs were compared using the Cramér-von Mises test, implemented in SALib. This procedure highlights key directions for revising experimental setups, possibly focusing on parameters which variations produce desirable or undesirable frequency distributions to enhance structural behavior of the penstock. A minimal sensitivity allows for streamlined efforts towards influential factors, simplifying experimental designs and improving overall efficiency. The results include the following observations.

• The RSA for $f_{1,1}$ (Figure A2. 1a) confirms the dominant influence of span length (L) and radius (R) on the model's behavior. L is particularly influential at the lower and higher frequency percentiles (<25 % and around 75 %), while R a notable impact across a broader frequency range. The other parameters exhibit minimal relative influence across the output range, confirming their limited effect on the bending mode.
• The RSA for $f_{2,1}$ (Figure A2. 1b) reveals that the head (H) exhibits the highest sensitivity at low percentiles, with a sharp decrease towards the mid-range and some variability at higher percentiles. R shows variable sensitivity across the entire frequency range and particularly significant at the extremes. The span length (L) displays moderate sensitivity, particularly at the lower and higher percentiles, with relatively stable low influence in the mid-range. The other parameters show low sensitivity across all percentiles, suggesting a minor impact on the $f_{2,1}$ mode shape. However, I/I0 maintains low sensitivity throughout most of the range except at very high frequencies, where its influence becomes notable.
• The RSA for $f_{3,1}$ (Figure A2. 1c) reveals sensitivity profiles that closely mirror those observed for $f_{2,1}$. The head (H) exhibits the highest sensitivity at low percentiles 0-20th while R has the highest sensitivity in the 80th percentile, indicating its significant impact on high frequencies. The other parameters demonstrate minimal relative influence across the frequency range of this oval mode.

#### 3.6.4 Factor range variability influence

In this section, we aim to verify if varying the range selections of the structural factors can alter sensitivity indices and to understand how this variability influences outputs $f_{1,1}$, $f_{2,1}$, and $f_{3,1}$. By applying the extended MCF inversely (i.e. targetting the input space), we divide each parameter space into 20 intervals based on percentile values to map their spatial influence (Figure A2. 2). This procedure suggests directions for experimental setup revision, by focusing on the key parameters within their critical range to refine model accuracy, thereby simplifying the model and achieving more accurate SA results. The results include the following observations:

• For $f_{1,1}$ (Figure A2. 2a): Adjusting input factor ranges reveals specific sensitivity increases for L within lower (0–20 %) and around the 80th percentile regions, suggesting crucial model behavior adjustments within these intervals, while all other parameters (t, P, $N_{s}$, $I/I_{0}$) exhibit uniformly minimal influence across their variation ranges.
• For $f_{2,1}$ (Figure A2. 2b): RSA highlights R's significant influence at low and high variation values, with a persistent moderate sensitivity across much of its range. L shows moderate sensitivity at lower values (<20 % percentile). H exhibits increased sensitivity at the start of its variation space, suggesting significant impact of low head values on $f_{2,1}$. Other parameters show minimal impact across their ranges.
• For $f_{3,1}$ (Figure A2. 2c): RSA reveals almost the same factors sensitivity with $f_{3,1}$: the R's marked sensitivity in lower percentiles. Head H exhibits increased sensitivity at the start of its variation space. Other parameters show stable limited impact across their ranges.

## 4 Discussion

From a methodological perspective, this methodology combines uncertainty quantification, parameter screening, advanced GSA techniques, and regional sensitivity analysis. Each phase contributes to a refined understanding of the model's sensitivities. While GSA applications in hydropower infrastructure are still limited, this study can serve as a foundation for prioritizing research efforts, refining design parameters, and tailoring impact assessments to specific hydropower systems.

A key consideration throughout the process was the balance between computational efficiency and model fidelity. By employing a phased approach—starting with broad uncertainty quantification and narrowing down to more detailed regional analysis—this methodology ensures that computational resources are allocated efficiently. Early use of screening methods significantly reduced the number of parameters, making it feasible to apply more computationally intensive methods, such as EFAST, to the high-impact parameters.

The process also unfolds over a temporal scale, as uncertainties evolve throughout the lifecycle of the penstock. In the early phases, structural parameters such as wall thickness, span length, and material properties dominate uncertainties. As the penstock ages, factors such as material fatigue and water pressure fluctuations become critical. This temporal evolution highlights the need for continuous sensitivity assessments to ensure reliable long-term operation. The structured methodology presented addresses these evolving uncertainties, supporting informed decision-making and effective maintenance strategies.

The ECDFs analysis plays a crucial role in providing detailed view of the distribution of model outputs, allowing for a better understanding of uncertainties and nonlinear behaviors in the penstock structure. These findings influence the interpretation of the model's reliability in several ways. First, the ECDFs help identify critical output ranges where unexpected behaviors may occur. For example, by pinpointing high-density zones or peaks in the distributions of natural frequencies, it becomes possible to determine where resonance phenomena are most likely. This gives clear indications of potential structural failure or excessive deformation risks, which is crucial for assessing the model's robustness against parameter variations and operational uncertainties. Moreover, this analysis enables the establishment of performance thresholds that, when exceeded, could compromise system reliability. Specifically, the ECDFs results provide probabilities associated with extreme values of the model's responses, such as critical frequencies or maximum deformations. This enhances the model's ability to anticipate extreme scenarios, ensuring that design decisions consider not only normal conditions but also unforeseen situations.

From an operational and future design perspective, the ECDF results can guide more informed decisions regarding design, reinforcement sizing, or internal pressure management. For instance, if the analysis reveals that certain geometric variations significantly increase the system's sensitivity to resonance or deformations, this may encourage adjustments in structural dimensions or the addition of damping devices to mitigate these effects. Additionally, identifying parameters with marginal influence on output distributions allows for design simplification without compromising safety or reliability, thereby making the structure more cost-effective.

The Morris method, employed for its efficiency in screening, is not designed to capture higher-order interactions in non-linear models. However, this does not impact the overall results, as Morris was used specifically to reduce the model's dimensionality, allowing more computationally intensive methods to be applied in the subsequent phase.

While this study demonstrates the robustness of the proposed GSA approach, it is important to recognize the limitations inherent in the individual methods used. The limitations of the Morris method in capturing interactions primarily stem from its OAT experimental design. While the extended version introduces improvements by refining the sampling process, it still falls short in accurately quantifying complex interactions between input variables. The Morris method provides an approximation of interactions through the variance of the elementary effects. This is achieved through the variance analysis of Morris trajectories, where significant variation in the results indicates the presence of potential interactions does not quantify interaction effects as precisely as other methods.

Moment-dependent methods, like variance-based sensitivity indices, focus on specific moments of the output distribution to quantify input uncertainties. While widely used for their simplicity and powerful in capturing parameter interactions, these methods assume that variance alone captures uncertainty, which can be misleading when the output is skewed or multi-modal. For instance, variance-based methods may inaccurately rank inputs or provide contradictory conclusions as they overlook important interactions within the complex output distribution. In contrast, moment-independent methods, such as the PAWN approach, evaluate sensitivity by considering the entire output distribution function. This enables a more complete understanding of how uncertainties affect the model, especially with complex outputs. Such method also stands out for its ability to use “recycled” samples, meaning it can operate on pre-existing datasets without requiring a specialized experimental design. It can operate on a given dataset, making it both computationally efficient and fast. It effectively captures input effects even in scenarios with extreme values or specific regions of interest, offering greater flexibility and accuracy. However, while Moment-independent methods offer a broader view of uncertainty and being particularly effective in non-linear models, they can require sometimes large sample sizes for accurate results, and can be less intuitive, making them challenging in fast-paced decision-making. By strategically combining these methods with others, the presented framework maximizes their strengths and overcome these limitations, resulting in a robust and efficient analysis.

The findings of the implemented application are inherently shaped by the limited uncertainty sources considered. In real-world applications, additional factors such as material properties, construction tolerances, and environmental forces must be accounted for to achieve a more comprehensive evaluation of penstock behavior. Moreover, correlations between parameters, which were treated independently in this analysis, should be further explored to capture the complex interdependencies often present in practice. The exploration of stiffener parameters in this application was limited. The number and shapes of stiffeners considered were constrained, which may have restricted a fuller understanding of their influence on structural integrity. Future research should expand the scope of stiffener modeling, incorporating a wider range of configurations to provide deeper insights into their role in enhancing the overall system's performance.

Finally, future research could explore the use of surrogate models [77] for large-scale penstock systems, providing an efficient alternative by approximating complex behaviors of the full-scale model with significantly lower computational cost. These models could enable more frequent and cost-effective sensitivity analyses without sacrificing accuracy. This step is particularly advantageous when the model requires long execution times, as is often the case in hydropower infrastructure simulations involving coupled structural dynamics and computational fluid dynamics (CFD).

On another note, while Finite element Modeling (FEM) have proven essential for analyzing the vibrational behavior of penstocks, as outlined in engineering standards such as the ASCE design guideline for penstocks [67], there remains potential to refine the accuracy of dynamic response predictions through the integration of data-driven identification techniques. Methods such as Observer/Kalman Filter Identification (OKID) and Numerical Subspace State-Space System Identification (N4SID), as demonstrated by Pappalardo et al. [78], offer systematic approaches to identify dynamic parameters using a combination of numerical and experimental data, potentially enhancing the robustness and precision of the analyses. Future research could further explore advanced damping techniques for penstock structures, building on methodologies such as those developed by Ref. [79]. In future research, extending the current work by incorporating these advanced system identification methods could provide a deeper understanding of the dynamic behavior of penstocks under real operating conditions.

## 5 Conclusion

This study presents a comprehensive methodology for conducting a GSA on structural penstock models, addressing challenges of high dimensionality, complexity, and non-linearity. Demonstrated through a prestressed modal analysis application, the methodology investigates the sensitivity of prominent mode shapes to various structural parameters.

Integrating UA and GSA, the research provides a systematic, step-by-step approach that enhances our understanding of penstock dynamics and offers a robust framework for addressing uncertainties in penstock design. The key steps include.

• An in-depth UA discerns the model's inherent variabilities, setting the stage for focused and impactful subsequent analyses. This phase involves systematic propagation of factor uncertainties through the structural model, determining the range and distribution of potential mode frequencies, and assessing the probabilities of exceeding critical vibrational thresholds through ECDFs. This provides a robust framework for risk assessment and management in penstock design.
• Initial sensitivity insights were extracted from scatter plot analyses, revealing key trends and hinting at potential complex interactions between the parameters. This LSA method sets the stage for a global perspective and more targeted SA to capture the intricate interactions and non-linear effects inherent in these structures.
• In the screening process, the extended Morris method was utilized to streamline the model by identifying less impactful factors, facilitating resource-efficient analyses without compromising the accuracy of the model. Four GSA methods were employed to examine key parameter effects across the entire input space, uncovering the direct impacts of individual parameters and intricate interactions. These methods include the EFAST, RBD-FAST for variance-based methods, and DMIM and PAWN method for density-based analysis. These methods were strategically selected and applied to ensure the methodological robustness of our sensitivity indices, with a convergence study validating the accuracy and reliability of the results. GSA findings underscore the impact of key parameters, including the wall thickness, the span radius and length, the head and slope, and some stiffener parameters, in determining their natural characteristics.

• The culmination of the workflow involved applying RSA to segmenting the response space to evaluate structural factor influences, ensuring practical alignment with optimization objectives. The RSA mapped the spatial distribution of parameter influence, providing insights into the system's behavior and suggesting targeted parameter management for optimization.

The practical implications are significant, enabling engineers to target design modifications for enhanced stability and performance of penstocks. The iterative nature of the proposed workflow ensures that models remain adaptable and responsive to new insights, supporting continuous optimization efforts in hydropower engineering. By identifying and prioritizing the most influential parameters, this methodology provides a valuable tool for engineers and researchers, driving forward the development of safer, more reliable, and economically efficient hydropower infrastructure.

Future research should extend this methodology to more complex models, incorporating additional environmental and operational variables. Additionally, integrating real-world observational data can further refine these models, enhancing their predictive accuracy and reliability. Inputs of interest for future SA may include model parameters, forcing variables, boundary and initial conditions, and choices of model structural configurations. As GSA continues to evolve in structural dynamics applications, this work provides a solid foundation for developing more resilient and efficient hydropower systems. Additionally, a detailed SA incorporating material properties, loading conditions, and boundary conditions should be conducted to precisely evaluate the natural frequencies and potential vibrations of specific penstock designs.

## CRediT authorship contribution statement

**Manal Haddouch:** Writing – review & editing, Writing – original draft, Visualization, Software, Methodology, Investigation, Formal analysis, Data curation, Conceptualization. **Imane Hajjout:** Writing – review & editing, Visualization, Software, Methodology, Investigation, Conceptualization. **El Mostapha Boudi:** Writing – review & editing, Visualization, Validation, Supervision, Methodology, Investigation, Conceptualization.

## Data availability statement

Data will be made available on request.

## Funding statement

This research did not receive any specific grant from funding agencies in the public, commercial, or not-for-profit sectors.

## Declaration of competing interest

The authors declare that they have no known competing financial interests or personal relationships that could have appeared to influence the work reported in this paper.

## Figures and Tables

**Fig. A.1.1:**
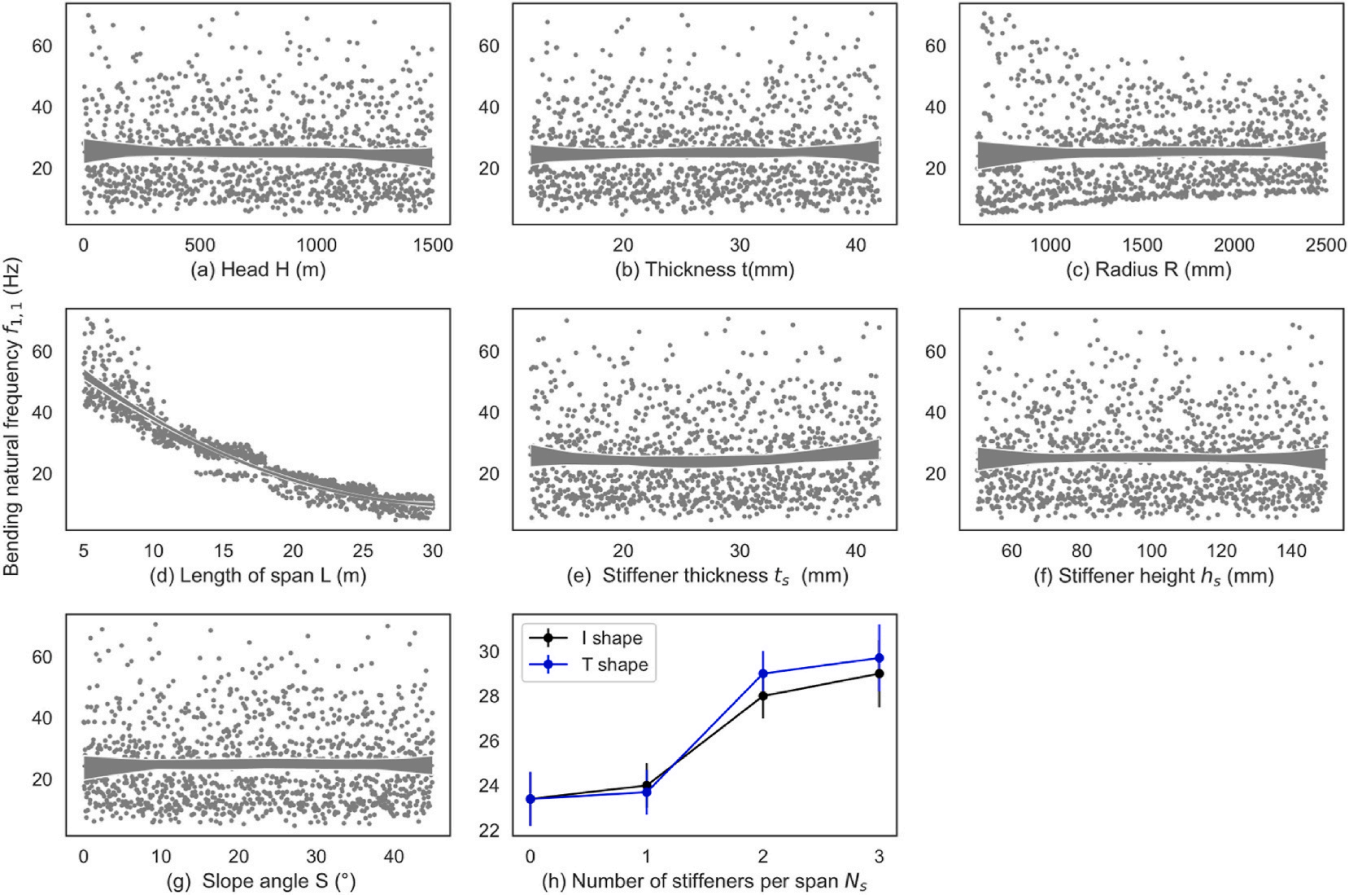
Correlations between structural parameters and the bending mode shape $f_{1,1}$): Scatter plots (a-g) for continuous parameters. Sparse scatterings indicate low sensitivity, while trends suggest significant influence. Point plot (h) illustrates the impact of adding stiffeners and their shapes.

**Fig. A.1.2:**
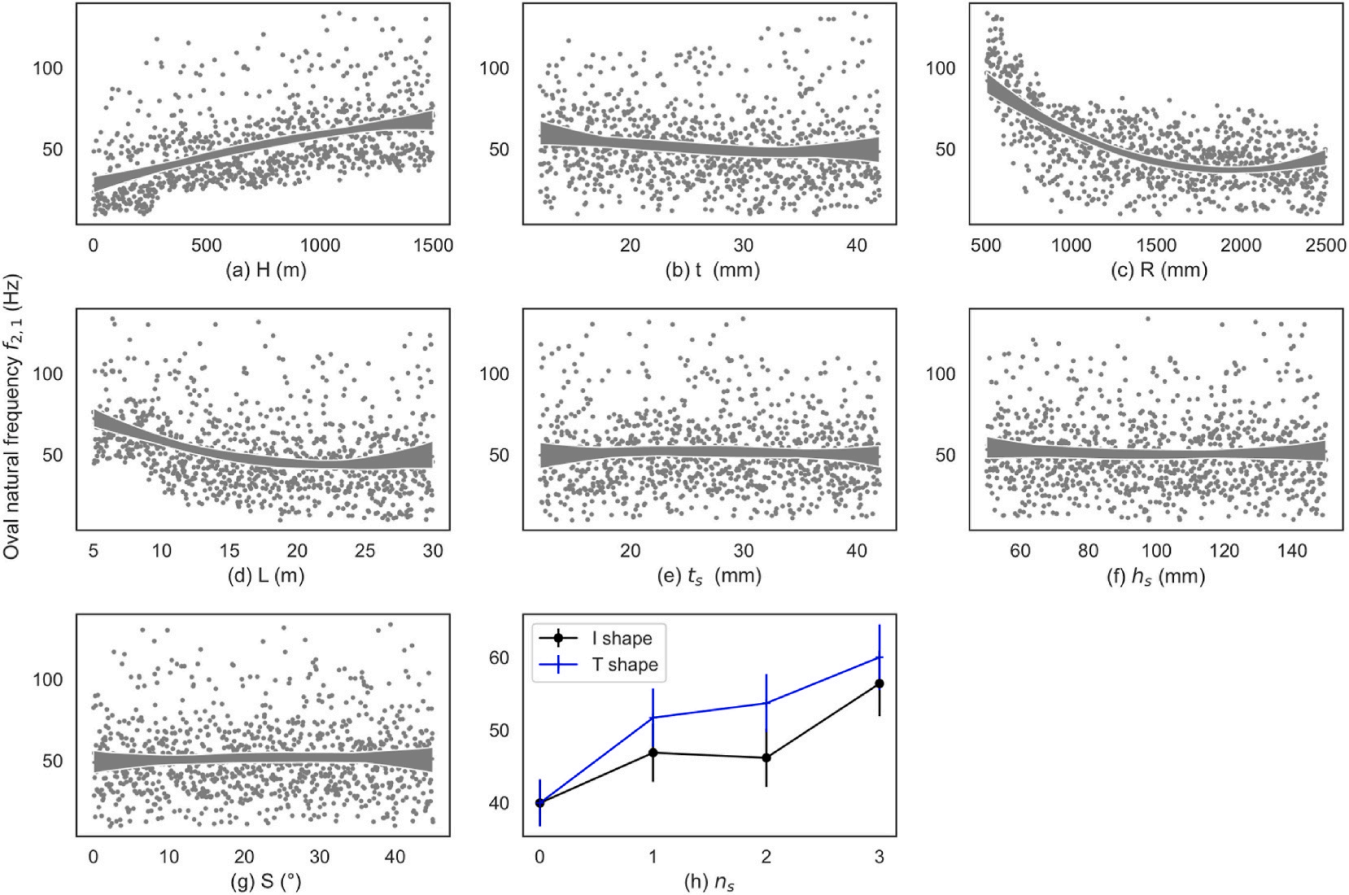
Relationship between structural parameters and the oval mode $f_{2,1}$, using scatter plots for continuous parameters (a-g) and a point plot for categorical parameters related to stiffener configurations (h).

**Fig. A.1.3:**
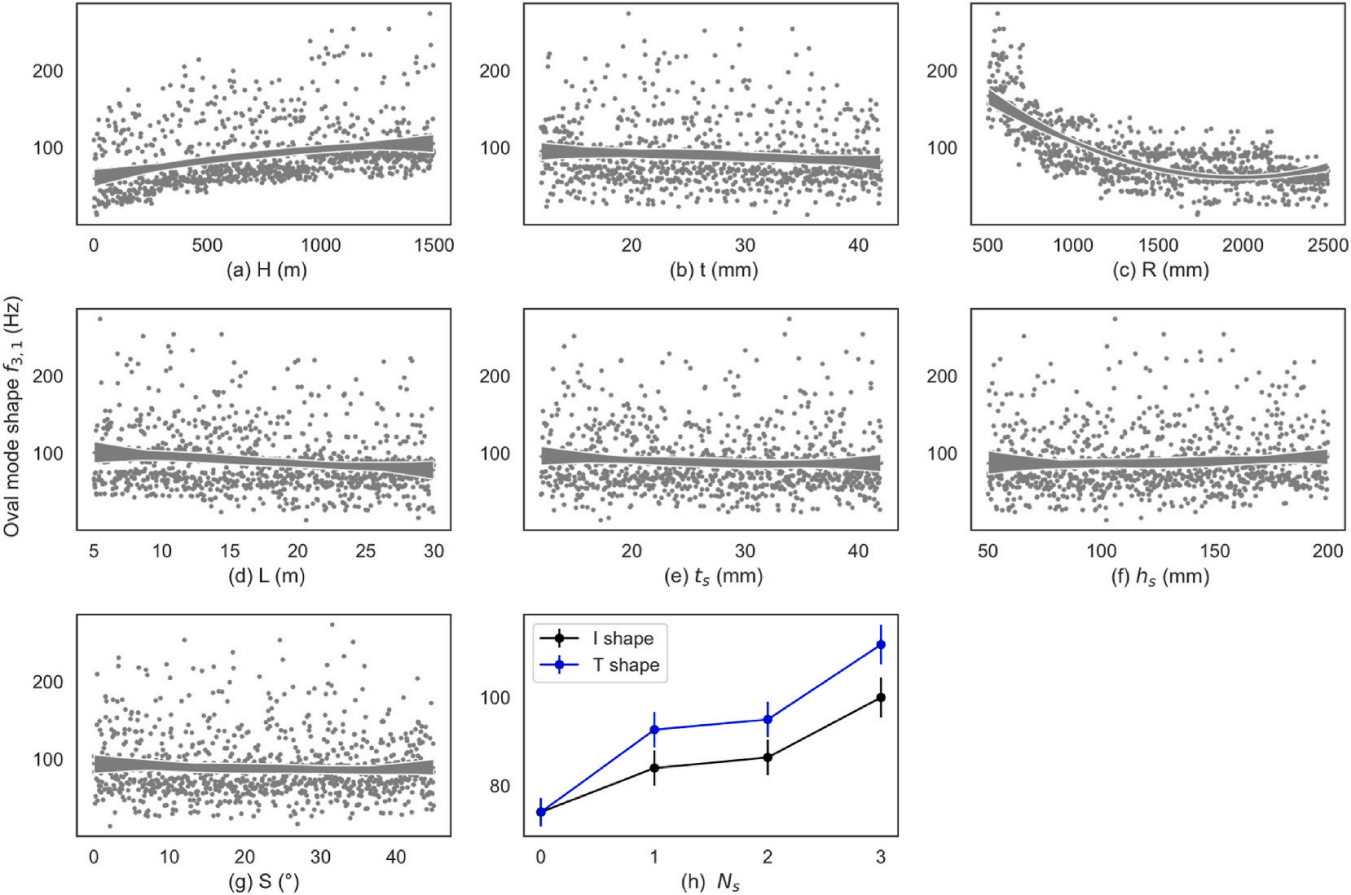
Scatter plots (a-g) and point plot (h) illustrating the relationships between structural parameters and the oval mode $f_{3,1}$.

**Fig. A.2.1:**
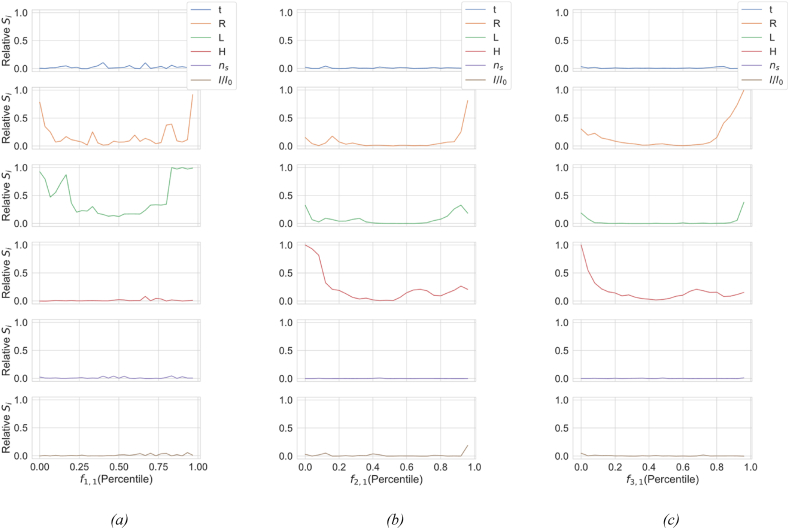
Regional sensitivity analysis using MCF approach, segmenting the mode frequency space into equal output sample groups and highlighting parameter influences across frequency percentiles for: (a) $f_{1,1}$, (b) $f_{2,1}$, (c) $f_{3,1}$.

**Fig. A.2.2:**
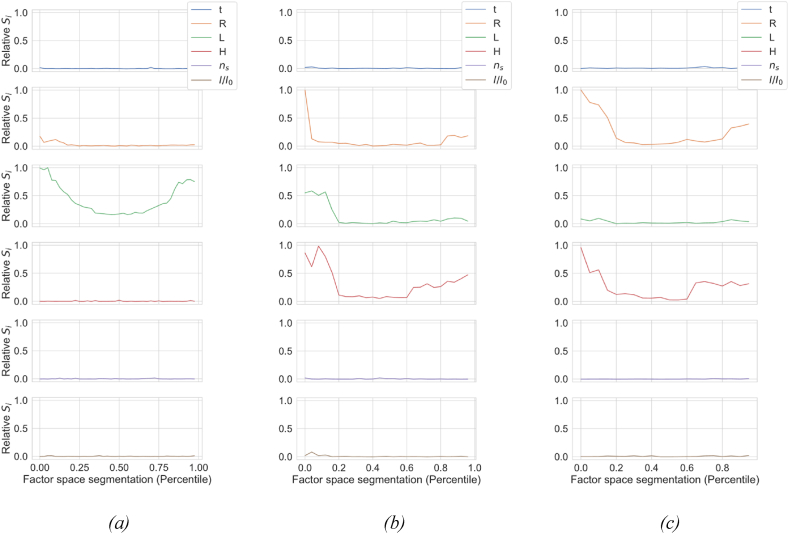
Regional sensitivity analysis using MCF approach inversely, dividing each parameter space into 20 intervals based on percentile values to map their influence for: (a) $f_{1,1}$, (b) $f_{2,1}$, (c) $f_{3,1}$.
